# Computational Insight into Protein Tyrosine Phosphatase 1B Inhibition: A Case Study of the Combined Ligand- and Structure-Based Approach

**DOI:** 10.1155/2017/4245613

**Published:** 2017-12-26

**Authors:** Xiangyu Zhang, Hailun Jiang, Wei Li, Jian Wang, Maosheng Cheng

**Affiliations:** ^1^Key Laboratory of Structure-Based Drug Design & Discovery, Ministry of Education, Shenyang Pharmaceutical University, Shenyang 110016, China; ^2^Faculty of Pharmaceutical Sciences, Toho University, Miyama 2-2-1, Funabashi, Chiba 274-8510, Japan

## Abstract

Protein tyrosine phosphatase 1B (PTP1B) is an attractive target for treating cancer, obesity, and type 2 diabetes. In our work, the way of combined ligand- and structure-based approach was applied to analyze the characteristics of PTP1B enzyme and its interaction with competitive inhibitors. Firstly, the pharmacophore model of PTP1B inhibitors was built based on the common feature of sixteen compounds. It was found that the pharmacophore model consisted of five chemical features: one aromatic ring (R) region, two hydrophobic (H) groups, and two hydrogen bond acceptors (A). To further elucidate the binding modes of these inhibitors with PTP1B active sites, four docking programs (AutoDock 4.0, AutoDock Vina 1.0, standard precision (SP) Glide 9.7, and extra precision (XP) Glide 9.7) were used. The characteristics of the active sites were then described by the conformations of the docking results. In conclusion, a combination of various pharmacophore features and the integration information of structure activity relationship (SAR) can be used to design novel potent PTP1B inhibitors.

## 1. Introduction

Diabetes mellitus has grown up to be a serious health problem around the world [1]. According to the World Health Organization (WHO), 422 million people around the world suffered from diabetes in 2016, up from 108 million people in 1980, and its prevalence is projected to be 764 million by 2030 [2]. The majority of these people suffered from type 2 diabetes (T2D), whose cause is insufficient insulin secretion in peripheral tissues [3]. Type 2 diabetes is extraordinarily associated with a variety of severe complications such as cardiovascular, eye, kidney, and nervous system diseases and diabetic nephropathy [1]. There are numerous oral diabetes medicines approved by the FDA, such as Invokana, Lyxumia, Nesina, and even Glucophage. Although great efforts have been made in this field, the therapeutic efficacy of market products is greatly limited by serious side effects and complicated drug-drug interactions in combination therapy. To solve these intractable problems, the main direction is to still search for new therapeutic agents [4]. Protein tyrosine phosphatase 1B (PTP1B), a negative regulator of insulin and leptin signaling pathways, is a promising target for the development of type 2 diabetes treatment.

Protein tyrosine phosphatases (PTPs) are a large family of enzymes that remove phosphate groups from phosphorylated tyrosine residues in various signal transduction pathways [5–9]. The PTPs were mainly characterized by an 11-residue signature sequence (I/V) HCXAGXXR (S/T/G), which is known as the PTP loop. PTP1B, the first non-receptor-bound protein tyrosine phosphatase isolated, is the best-studied member of humans. Since its discovery more than 25 years ago, PTP1B has proved to play a critical role in multiple cellular processes, especially glucose uptake, body mass regulation, motility, and proliferation [10, 11]. Tahtah et al. [2] and Klaman et al. [12] have reported that PTP1B knockout mice had an increased insulin sensitivity through improved glucose clearance and increased resistance to diet-induced obesity without any phenotypic abnormalities. Some studies suggested that PTP1B inhibitors could reduce obesity [2] and the X-linked neurological disorder Rett syndrome (RTT) [13].

**Figure 1:**
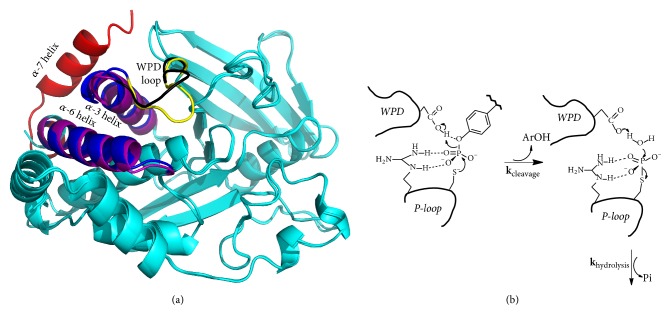
(a) Structure of PTP1B labeled with significant regions (3EAX superimposed with 2QBP). The WPD (closed, colored by yellow) was shown in blue with *α*-3 and *α*-6 helices, and the *α*-7 helix was shown in red. The WPD (open, colored by black) was shown in purple with *α*-3 and *α*-6 helices at the inactive state. (b) PTP catalytic reaction of cleavage and hydrolysis.

To date, numerous potent PTP1B inhibitors have been reported in literatures [14–19], and they could be classified into two major types: noncompetitive and competitive inhibitors. X-ray crystallographic analysis revealed that noncompetitive inhibitors occupied and interacted with the enzyme active site or allosteric binding pocket ~20 Å away from the catalytic site (helices *α*3, *α*6, and *α*7) [14]. These compounds could stabilize PTP1B in the inactive conformation when WPD loop opens (Figure 1(a)) [15]. Importantly, noncompetitive inhibitors could make cells permeable and enhance insulin signaling in hepatoma cells [15]. However, contributions of these molecules were very limited, because they had only micromolar affinity with IC_50_ values in the 10–100 *μ*M range. As for competitive inhibitors, they were accompanied by a rotation of the WPD loop from an open (hydrolysis incompetent) to a closed (hydrolysis competent) position (Figure 1(a)) [19]. It was reported that the isothiazolidinone (IZD) derivatives had high activities, and the best value of IC_50_ was 190 nM [16], which was a ~50-fold improvement in potency compared with the noncompetitive inhibitors. Upon further exploration, the best of thiophene-2-carboxylic acid derivatives had subnanomolar activities with a Ki value of 0.68 nM [18], which was 14,000-fold more potent than the noncompetitive counterpart. Existing PTP1B competitive inhibitors cannot meet the requirements of cell membrane permeability, and very few of the products could be finally applied in the clinical treatment. Thus, structure-based drug design and discovery strategy should be developed to facilitate the clinical efficacy by further improving drug properties.

In a word, it was essential to comprehensively understand PTP1B's active sites and its competitive inhibitors. In this work, we constructed the common feature pharmacophore model of PTP1B competitive inhibitors and estimated protein-ligand binding affinities by different docking protocols. PTP1B crystal structures were then analyzed to reveal the properties of binding sites. To our knowledge, this is the first time that systematical combinations of ligand-based and structure-based approaches provide an insight into PTP1B's active site and the interaction with its ligands by computational simulation. This method provided a significant strategy for PTP1B inhibitors' study.

## 2. Computational Methods

All calculations were handled on a Dell PowerEdge R910 workstation. Chemical structures were prepared by SYBYL 6.91 (Tripos Inc.), the pharmacophore was generated in the Discovery Studio 3.0 software package (BIOVIA Inc.), and docking studies were performed with AutoDock 4.0, AutoDock Vina 1.1, standard precision (SP) Glide 9.7, and extra precision (XP) Glide 9.7 in Schrödinger software.

### 2.1. Preparation of Proteins and Ligands

The 16 inhibitors were sketched and optimized in SYBYL 6.91 with Tripos force field and Gasteiger–Hückel charges, utilizing the Steepest Descent algorithm, followed by the Conjugate Gradient and Adopted Basis Newton-Raphson algorithms, with convergence gradient values of 0.1 kcal*∗*mol^−1^, 0.01 kcal*∗*mol^−1^, and 0.001 kcal*∗*mol^−1^, respectively [20]. Other parameters were set as default.

There were 7 experimental crystal structures of PTP1B downloaded from the RCSB Protein Data Bank (PDB codes: 2CM7 [16], 2CMA [16], 2CMB [16], 2CNG [21], 2QBP [18], 2QBQ [18], and 2ZN7 [22]) (http://www.rcsb.org/pdb/). The seven proteins were prepared by Discovery Studio 3.0 software package. The missing amino acid residues were built and hydrogen atoms were added to the protein. All water molecules were removed from structures and then loop segments were completed.

Since PTP1B inhibitors were flexible molecules with similar structures, the docking experiment was also performed by Glide in Schrödinger (version 2014). And key amino acids were set as constraints to ensure their participation in hydrogen bond interactions. Protein and ligand structures were input as complete all-atom 3D structures with a reasonable geometry for Glide. The PTP1B cocrystallized structure was processed with the protein preparation wizard in Schrödinger suite. Protein integrity was checked to correct structure defects and prepared by adding hydrogen atoms, deleting solvent water molecules, and defining right bonds orders. Amino acids such as Asp, Lys, and His were assigned as protonated and tautomeric at pH 7.4. Afterwards, all hydrogen atoms of PTP1B complexes were optimized with OPLS_2005 force field, which minimized and converged heavy atoms to an RMSD of 0.3.

### 2.2. Generation of Common Feature Pharmacophore

The common feature pharmacophore module in Discovery Studio 3.0 was used to create pharmacophores automatically with 16 prepared compounds. In this work, five chemical feature types, that is, aromatic ring R, hydrophobic group H, ionizable positive center P and negative center N, hydrogen bond acceptor A, and donor D, were included for feature mapping. Parameters were set up by the following: maximum pharmacophore hypotheses were set to 15; the values of minimum features and maximum features were 4 and 6, respectively; the maximum distances of charge, hydrogen bond, and hydrophobic and exclusion volume were defined as 5.6, 3.0, 5.5, and 5.0, respectively. Other parameters were set as default in Discovery Studio 3.0.

### 2.3. Validation of the Pharmacophore

Further validation of the pharmacophore hypothesis was assessed by fit values of a decoy set comprised of another 49 PTP1B inhibitors with 25 reported PTP1B inhibitors and 24 inactive compounds. The decoy set was mapped onto the pharmacophore models by Ligand Profiler in DS 3.0. Results of the model mapping onto inhibitors and Heatmap showed the most relevant pharmacophore model, where the active inhibitors could be distinguished from the inactive ones based on fit values. Finally, the selected pharmacophore hypothesis was utilized to match some chemical structures correlating to the SAR.

### 2.4. Molecular Docking

Computer‐aided docking is a suitable probe for explaining the receptor-ligand interactions valid in the drug discovery. This method was used to predict the placement, binding affinity, and inhibitors activity of cocrystallized ligands in the binding pocket of PTP1B. The optimized parameters of four docking programs were listed as follows.

AutoDock and AutoDock Vina implemented a Lamarckian Genetic Algorithm (LGA) [23]. The seven crystal structures of PTP1B have already been prepared by DS 3.0. AutoDock Tools 1.5.4 (ADT) was used to prepare input PDBQT files and to calculate a grid box. As for AutoDock, the grid map consisted of 80 Å × 80 Å × 80 Å points around the active site, with a grid spacing of 0.375 Å. Afterwards, the center of the grid was set to each receptor at the centroid of their associated reference ligands. The protocol involved a maximum number of energy evaluations of 25,000,000, and the number of iterations was 3000 and 100 conformations were generated. All other parameters were set as default. All the docking poses were clustered together based on RMSD in which differences were less than 2.0 Å. The conformations were selected as representative, which had the most favorable free energy or the highest percentage frequency. AutoDock Vina was also used to dock and predict the binding affinity (kcal/mol) of all training compounds. Finally, theoretical results of the molecular docking were compared with the experimental antibacterial data of tested compounds.

As for Glide docking, crystal structures of PTP1B should be prepared by the protein preparation wizard in Schrödinger suite. Afterwards, receptor grids were generated before docking with the active site determined by the position of cocrystal ligand. Crystal structures of PTP1B (PDB code: 2CM7, 2CMA, 2CMB, 2CNG, 2QBP, 2QBQ, and 2ZN7) were imported into Glide 9.7, defined as the receptor structure and the location of active site with a box of size 13 Å × 13 Å × 13 Å. The OPLS_2005 force field was used for grid generation [24, 25]. The standard precision (SP) and the extra precision (XP) protocols were set for docking studies with two crucial residues, Lys120 and Arg221, in constrained binding to get accurate results. All other parameters were maintained as default.

Discovery Studio 2017 Client was used for molecular interaction analysis. To improve the accuracy of molecular docking calculation, Xscore was utilized to predict the binding free energies of compounds with PTP1B in this study.

## 3. Results and Discussion

### 3.1. Generation of Common Feature Pharmacophore

**Figure 2:**
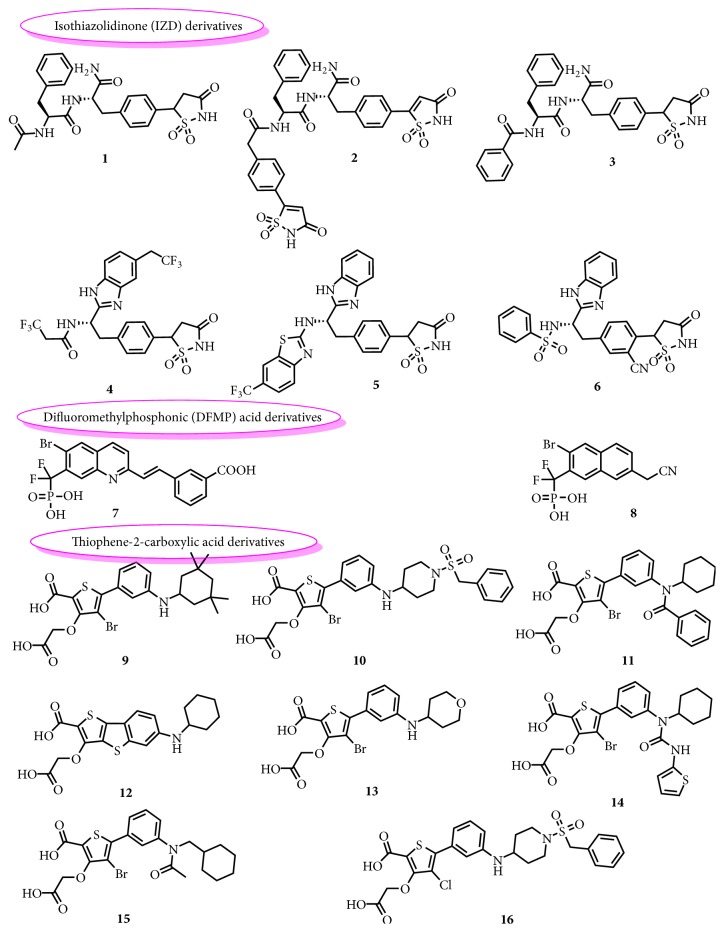
Chemical structures of representative isothiazolidinone derivatives (**1**–**6**), difluoromethylphosphonic acid derivatives (**7**-**8**), and thiophene-2-carboxylic acid derivatives of PTP1B inhibitors (**9**–**16**) considered as training set molecules for the pharmacophore model.

As the pharmacophore model required, molecules should have a large variety in chemical space and interact through a similar binding mechanism with the target protein [23]. In both major types of PTP1B inhibitors, the competitive inhibitors of higher activities (Table 1) and selectivity were chosen for analysis. The three main families of representative compounds were selected in Figure 2, including (i) isothiazolidinone (IZD) derivatives [16, 21], (ii) difluoromethylphosphonic (DFMP) acid derivatives [17], and finally (iii) thiophene-2-carboxylic acid derivatives [18, 22, 26].

On account of diversities and activities of these structures (Figure 2), the common feature pharmacophores were generated to describe SAR. Sixteen inhibitors were submitted as the training set. These compounds were sketched and optimized in SYBYL 6.91 and 3D feature-based alignments were provided by DS 3.0 software.

As shown in Table 2, fifteen pharmacophore hypotheses ranged from 82.987 to 96.526 with five chemical features. Based on differences between the chemical feature and component, these hypotheses were classified as three groups: RNHHA (01, 03, 05, 08, 11, 13), RHHAA (02, 04, 06, 07, 10, 12, 15), and HHHAA (09, 14).

### 3.2. Validating the Pharmacophore Model

**Figure 3:**
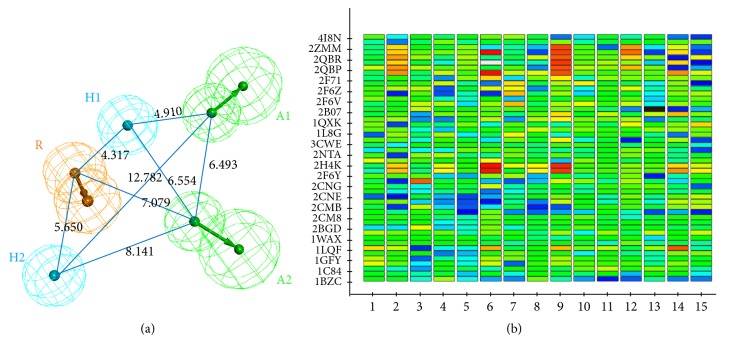
(a) Selected pharmacophore hypothesis 06 for PTP1B inhibitors consisting of two hydrogen bond acceptors A (green), two hydrophobic groups H (cyan), and an aromatic ring R (orange). Distances between the features were expressed in Å, with a tolerance sphere of radii ±0.8 Å. (b) Heatmap of Ligand Profiler revealed the best pharmacophore of hypothesis 06 by scaling the fit values.

In order to validate the reliability of pharmacophore hypotheses, a decoy set was prepared. It consisted of 49 compounds which contained the 25 known active inhibitors of PTP1B [16, 18, 21] and the other 24 inactive compounds. The excellent pharmacophore was able to distinguish between active and inactive compounds. The decoy set was mapped onto all the 15 hypotheses. The results of the test were shown by Heatmap (Figure 3(a)). Heatmap was a plot where the fit values were represented in a two-dimensional color map. Light green showed the fit values of compounds more than 2; otherwise, it was dark blue or even black. Therefore, analyses of these Heat Maps indicated that the sixth hypothesis (RHHAA) was considered as the most relevant one among the fifteen pharmacophore hypotheses (Figure 3(b)).

**Figure 4:**
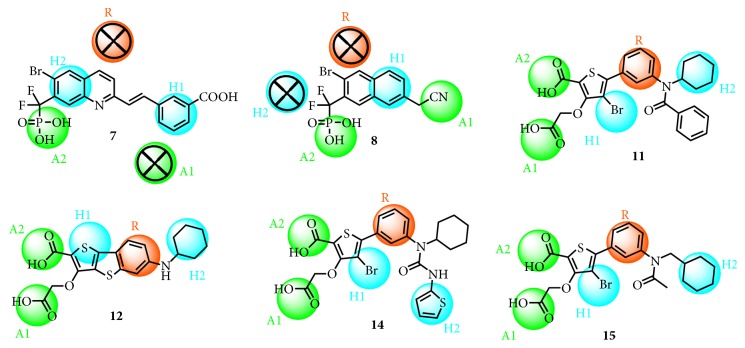
2D mapping of the pharmacophore features onto compounds representative of each family. The features were color-coded as follows: hydrophobic group (H) in cyan, aromatic ring (R) in orange, and hydrogen bond acceptor (A) in green. An attached × indicates that a feature was not mapped.

In order to describe SAR more accurately, two representative molecules were picked up among three types of PTP1B inhibitors. The six representative inhibitors were mapped onto the representation of hypothesis 06 (Figure 4). As illustrated above, A1 and A2 involved phosphate group, carboxylic acid group, and isothiazolidinone. R was defined by pyridine, pyrazole, phenyl, and benzofuran moiety. The hydrophobic functions of H1 and H2 consisted of either aromatic moieties or halogen atoms, like hexamethylene, thiophene, bromine, and chlorine atoms. However, compounds** 7** and** 8**, the difluoromethylphosphonic acid (DFMP) derivatives, were smaller and more rigid than the other inhibitors and could not match R or A1. In general, the chemical features of H1, H2, and A2 were essential for all the representative inhibitors in 06 pharmacophore hypotheses.

To determine how well the pharmacophore fitted the active site of PTP1B, some reported X-ray complex structures of the PTP1B have been downloaded from PDB. Combining the 3D structure of PTP1B and the results of molecular docking would help us to improve the pharmacophore.

### 3.3. Molecular Docking

To date, seven classical crystal structures of human PTP1B-ligand complexes (PDB codes: 2CM7, 2CMA, 2CMB, 2CNG, 2QBP, 2QBQ, and 2ZN7) have been determined. Meanwhile, these ligands were used to conduct native docking to measure the docking conformations. Four different docking programs—AutoDock, AutoDock Vina [27], SP Glide [24, 28], and XP Glide [29, 30]—were used for improving the accuracy of prediction. Then, Xscore followed by molecular docking was reliable and accurate for forecasting protein-ligand binding free energies (Table 3).

The docking results were evaluated by comparing values of score energy and docking poses via AutoDock 4, AutoDock Vina, SP Glide, XP Glide, and Xscore. Through analysis of these results of native docking simulations, most binding energy scores could accurately forecast the ligand activities except for AutoDock Vina program. The lowest binding energy and the highest docking score demonstrated that these compounds (ligands) presented well favorable interactions between them and the human PTP1B (receptors). The application of various reliable docking protocols could achieve the accuracy of docking poses.

However, a severe problem was the docking accuracy of cocrystallized complexes which docked new ligands rather than self-docking [24, 25]. The great approach to test this was cross-docking; for the same target, there is usually more than one ligand-receptor complex; what we do is dock one's ligand into other complexes. Therefore, seven classical crystal structures of PTP1B complexes were aligned to the template protein (PDB code: 2CMB), which had the highest resolution among seven crystal structures. The root-mean-square deviations (RMSDs) of each ligand were calculated by comparing it to its position in the native protein structure to estimate the docking reliability. If the sampling algorithm could not avoid incorrect penalties in self-docking, it was probably not able to do so in a much more challenging cross-docking situation [24]. Thus, three reliable docking programs were utilized (Table 4).

**Figure 5:**
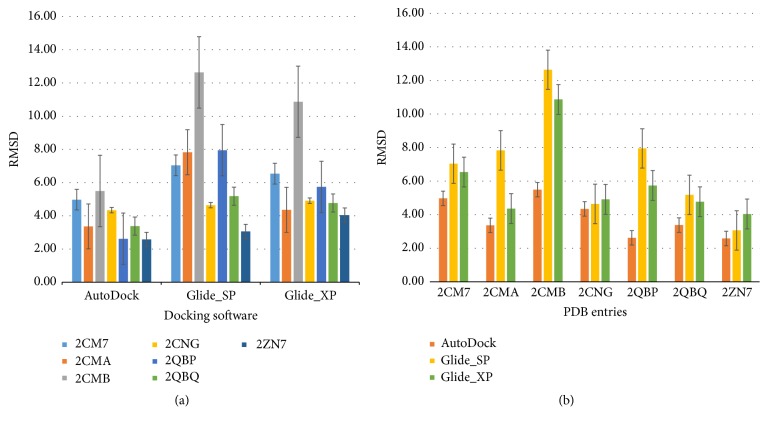
Analysis of cross-docking simulations. For each protein structure of PTP1B (PDB codes: 2CM7, 2CMA, 2CMB, 2CNG, 2QBP, 2QBQ, and 2ZN7), docked poses per ligand were selected to calculate the average RMSD.

To our surprise, the results of the cross-docking simulations revealed that all of the seven ligands of average RMSD were quite large.  Figure 5(a) shows a comparison of RMSDs for each docking protocol (AutoDock 4, SP Glide, and XP Glide) docked with all seven cocrystallized ligands. And in Figure 5(b), three docking programs were selected to calculate the average RMSDs per protein structure of PTP1B (PDB codes: 2CM7, 2CMA, 2CMB, 2CNG, 2QBP, 2QBQ, and 2ZN7). As for the cross-docking results of each protein generated by three docking programs, RMSD values were greater than 2. Thus, several proteins might not be clustered together.

**Figure 6:**
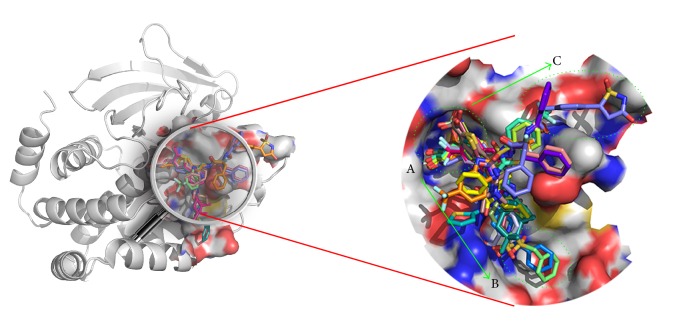
Tridimensional view of alignment of all seven cocrystallized ligands in PTP1B active site. Each molecule was shown as carbon in difference colors. PyMol was used to analyze the binding pattern. The A site was the positively charged catalytic phosphate-binding pocket; the B site was the secondary phosphate-binding pocket that played a role in substrate specificity; the C site was a third phosphate-binding site where negatively charged substituents could be accommodated in this large flat region. Arrows indicate that cocrystallized inhibitors bind in the A site and extend into the B and/or C sites.

The binding modes for the PTP1B active site and all cocrystallized ligands are shown in Figure 6. Interestingly, their binding patterns had a little distinction. According to the binding models of cocrystallized ligands in the PTP1B active site, they were classified into two groups: PDB codes 2CM7, 2CMA, 2CMB, and 2CNG and PDB codes 2QBP, 2QBQ, and 2ZN7. Four cocrystallized ligands (PDB codes: 2CM7, 2CMA, 2CMB, and 2CNG) bound in primary phosphate-binding pocket (A site [16]) and a large flat region (C site [21]) and the other ligands (PDB codes: 2QBP, 2QBQ, and 2ZN7) were described by extension of the molecule from the enzyme active site (A site) into the second phosphotyrosine binding site (B site [18]).

**Figure 7:**
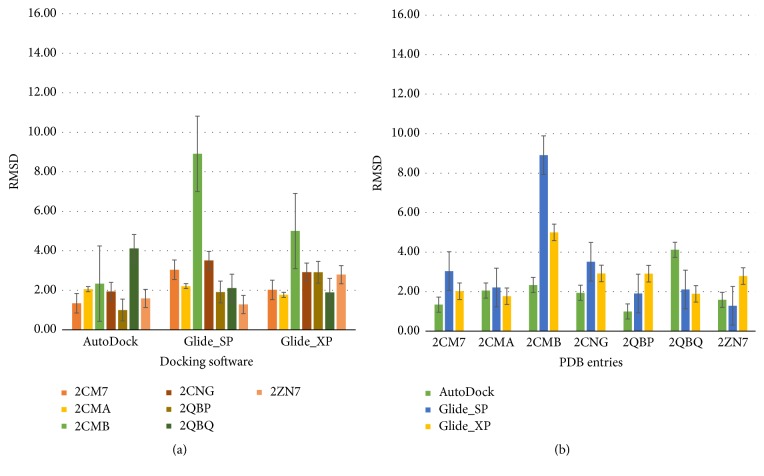
Analysis of cross-docking simulations by two groups. For each protein structure of PTP1B (PDB codes: 2CM7, 2CMA, 2CMB, 2CNG, 2QBP, 2QBQ, and 2ZN7), docked poses per ligand were selected to calculate the average RMSD.

Thus, cross-docking results should also be classified into two groups depending on their models of action. Results of new cross-docking are shown in Figure 7. The docked ligand of 2CMA had the minimum average RMSD from the first group, whereas the docked ligand of 2QBP had the minimum average RMSD from the second group. Thus, 2CMA and 2QBP were selected as standard templates to evaluate the training set and provide an insight into the active site of PTP1B.

### 3.4. Characterization of the PTP1B Active Site

Since the crystal structure of PTP1B was identified for the first time in 1994, a large amount of structural data has been reported [31]. Structurally, PTP1B was made up of 435 amino acids, but only three fragments with relatively short length (282, 298, or 321 residues) were typically considered for biochemical and biological studies [11]. According to crystallographic studies, PTP1B existed in two forms: open (inactive state) and closed (active state) [32]. Wiesmann et al. and Liu et al. reported that allosteric inhibitors could occupy an adaptable part and stabilize a conformation that was associated with the open form of PTP1B. Meanwhile, most competitive inhibitors bound effectively to the PTP1B active site in the closed form of PTP1B.

A wealth of structural data revealed that the active site could be separated into five subpockets in the protein (A, B, C, D, and E). Among them, the A, B, and C sites (Figure 6) were essential for protein function and conducted to regulate insulin signaling markers [16, 33, 34]. The primary phosphate-binding pocket was A site, where phosphotyrosine (pTyr) residues of the insulin receptor (IR) kinase activation peptide were dephosphorylated [35]. This pocket was not large with 10 Å width and 9 Å depth which were measured by length from Tyr46 to Gln262 and distance from Cys215 to Phe182, respectively [36, 37]. However, A catalysis site contained a large quantity of polar amino acids, like Asp48, Cys215, Ser216, Arg221, Gln262, and Gln266. So, the compounds which bound to it would have good potency as well as poor membrane permeability.

The B site was a secondary binding pocket, which bound with pTyr side chain by Arg254 and Arg24 on the surface of the protein. It was made up by a series of key residues, such as Arg254, Arg24, Met258, Val49, Gly259, Phe52, and Ile219. Compared to A site, it was shallower and larger in shape and noncatalytic in function. But Wilson and Wan et al. showed that small inhibitors could improve activities and selectivity by occupying this pocket. Thus, the B site was no doubt a significant active site.

Finally, the C site was also called the third phosphate-binding pocket, which is adjacent to the primary phosphate-binding pocket (A site). Structurally, the C site was a large flat region and was exposed to a quantity of polar solvent. Based on these properties, the C site could accommodate many negatively charged groups [16]. Some surrounding residues like Lys41, Tyr46, Arg47, and Asp48 played a key role in enhancing the biological activity of inhibitors [16, 21].

In a word, pockets of B and C surrounding the A site were explored for designing and optimizing potential candidates, which had greater selectivity, activities, and cell membrane permeability.

### 3.5. Inhibitor Binding Analysis

**Figure 8:**
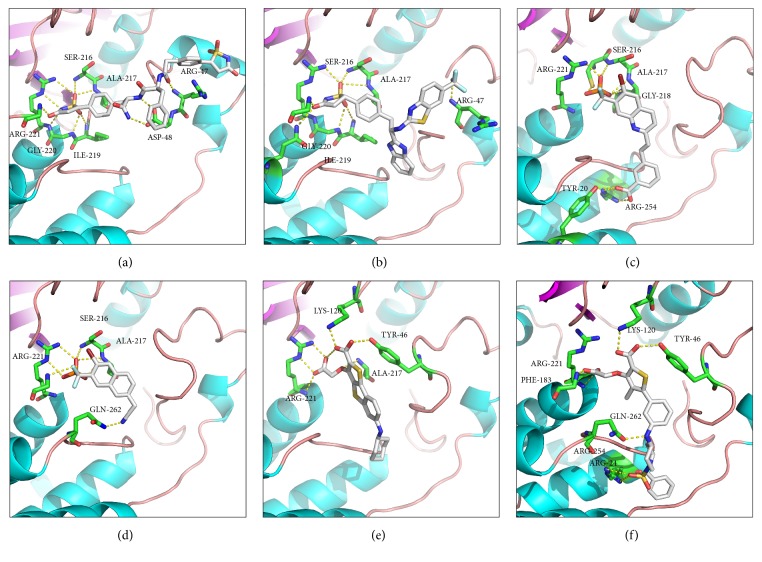
Docking of compounds** 3** (a),** 5** (b),** 7** (c),** 8** (d),** 12** (e), and** 16** (f) into the active site of PTP1B with key amino acid residues (colored by green) in represented ligand binding poses. The pictures were prepared by PyMol software.

The training set of PTP1B was predicted by molecular docking, and the docking scores of different programs are shown in Table  S1 (in Supplementary Material available online at https://doi.org/10.1155/2017/4245613). The least binding energy and the most rational binding pattern between the inhibitors and PTP1B were selected by the three docking protocols. And the nonbond interaction of each compound in PTP1B active sites is shown in Table  S2. As expected, isothiazolidinone derivatives (compounds** 1**–**6**) bound in the A site and the C site, validating the prediction by molecular docking with 2CMA. However, difluoromethylphosphonic acid derivatives (compounds** 7** and** 8**) and thiophene-2-carboxylic acid derivatives (compounds** 9**–**16**) were described by extension from the A site into the B site, which were proofed by docking with 2QBP. The binding modes of all 16 inhibitors were selected by AutoDock software. The binding pocket of the PTP1B and binding modes of each training set are shown in Figures  S1 and S2 in the Supplementary Material, respectively. Among the training set, six compounds were selected, which represented three types of inhibitors, including compounds** 3**,** 5**,** 7**,** 8**,** 12**, and** 16** (Figure 8).

From the docking results, one rational binding pattern was identified for compound** 3** binding into the A site and C site (Figure 8(a)). The heterocycle of isothiazolidinone bound at the center of the A site (Cys215–Arg221), which was in the vicinity of eight donors. Six amino acids (Gly220, Gly218, Cys215, Ile219, Arg221, and Ser216) bound to the sulfone oxygen and nitrogen anions. Meanwhile, two additional hydrogen bonds were depicted between the oxygen of Asp48 and hydrogen of the amides. The sulfone oxygen and hydrogen bound to Lys36 and Arg47 in the C site. The phenyl ring directly formed a hydrophobic interaction with Ala217, Ile219, and Phe182.

The major interactions between** 5** and PTP1B active sites are shown in Figure 8(b), which was similar to** 3**. However, it was noted that there is a hydrogen bond between the trifluoromethyl and Lys120 in the C site. Key interactions involved two strong hydrogen bonds between Tyr181 and nitrogen atoms of benziminazole and aliphatic amine. The two hydrophobic interactions were also observed between benziminazole and Val49 and between benzoxazole and Tyr46. The widespread network of hydrogen bonds and the interactions of key residues were the main reasons why IZD analogs (**3** and** 5**) were potent inhibitors.

DFMP derivatives (**7** and** 8**) displayed good activity (IC_50_ = 7 nM and 90 nM) for PTP1B (Figures 8(c) and 8(d)). The hydrogen of the phosphonic acid donated a nonclassic carbon H-bond to Phe182, Cys215, Ser216, Ala217, Gly218, and Arg221. Moreover, quinoline/naphthalene ring filled the hydrophobic pocket via Phe182, Lys120, Tyr46, and Val49. The binding modes for these compounds and PTP1B were beneficial to fit the active site regions and useful as templates to deeply develop more potent PTP1B inhibitors. Unfortunately, DFMP derivatives had bad physical and chemical properties, which was reported by experiment of oral bioavailability in rodents [17]. Therefore, it was apparent that these inhibitors should alter their polar residues or large lipophilic groups for enhancing oral bioavailability.

The lead compounds of** 12** and** 16** (Figures 8(e) and 8(f)) were started with a HTS campaign, reported by Moretto et al. [26]. Though the potency of the lead compound was weak (*K*_*i*_ = 230 *μ*M), the availability of structural information provided guidance for further optimization. Dramatic changes have taken place by using flexible linkers to bridge two fragments from site A to site B. The thiophene ring mimicked the phenyl ring of pTyr which provided *π*-*π* interactions with Tyr46 and Phe182. The carboxyl groups of acidic side chain formed a salt bridge with Arg221 and Lys120 in the A site. In addition, van der Waals interactions between the Met258 side chain and the cyclohexyl of** 12** played a key role in binding affinity. These were efficient manners to guide and design the novel inhibitors by reasonable SAR information.

### 3.6. Comparison of the Docking Results with the Pharmacophore Model: Towards an Interaction Model within the PTP1B Active Site

In an attempt to evaluate the pharmacophore model by molecular docking results, the competitive inhibitors in their bioactive conformation (bound to PTP1B) were aligned to the common feature pharmacophore model hypothesis. For all studied compounds except** 1**,** 2**,** 6**,** 7**, and** 8**, all docking poses were well overlaid. This observation proved that the proposed common feature pharmacophore model could fit the binding cavity of the A site and B site.

Through analysis of molecular docking results, it was identified that five pharmacophore points corresponded to highly conserved interactions with major residues in the catalytic site of PTP1B. Indeed, two hydrogen bond acceptors (A1 and A2) were located at the A site and interacted strongly with Lys120, Phe182, Tyr46, Ile219, and Arg221. The aromatic ring (R) was mapped by groups such as thiophene, quinoline, or tricyclic ring and formed an interaction with Ala217. A hydrophobic group H2 accommodated the small lipophilic groups enclosed by Ile219 and Ala217. However, the other hydrophobic group H1 was not mapped by docking poses of inhibitors, which was located at the C site. Consequently, it was suggested that this hydrophobic group was not an essential feature for optimal interaction with binding models of the A site and B site.

To refine the pharmacophore model, molecular docking results and structural information of PTP1B were merged in the pharmacophore generation. Based on binding models, the structures could be divided into two groups:** 1**–**6** were added to the first group and** 7**–**16** were allocated to the other group. Then, the docked poses of the two groups were imported into PTP1B active sites and the parameter of conformational generation was none, respectively. Fifteen new pharmacophore hypotheses of each group were generated with the same parameter setting as original common feature modeling. Fifteen pharmacophore hypotheses were ranged from 82.987 to 96.526 with five chemical features. The first group consisted of five chemical features and pharmacophore hypotheses were ranged from 73.824 to 84.469. The other mainly contained four or five chemical features and had scores ranging from 76.014 to 88.864.

**Figure 9:**
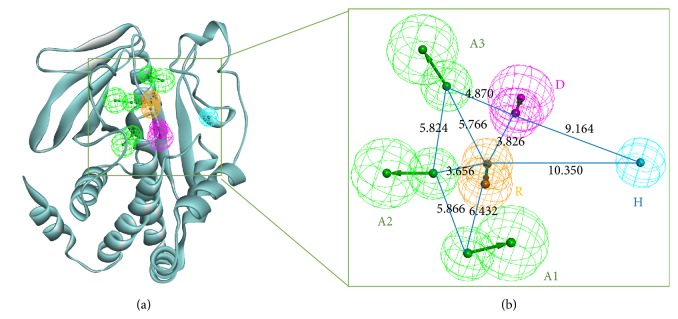
(a) Refined pharmacophore hypothesis superimposed onto the PTP1B active site. (b) Refined pharmacophore hypothesis. The pharmacophore features were as follows: hydrogen bond acceptors (A1, A2, and A3), hydrogen bond donors (D), hydrophobic features (H), and aromatic ring (R). Distances between the features are expressed in Å, with a tolerance of ±0.8 Å.

Deep analysis of new pharmacophore hypotheses revealed that the hypo 05 (RDAAA) gave the best correlation with the first group, and in the other group, hypo 05 (RHAA) was considered as the most relevant by superimposing onto the PTP1B active site. In order to better describe characteristics of the active site, two groups of pharmacophores were superimposed to obtain a refined pharmacophore (RHDAAA) (Figure 9). Among new refined pharmacophores, two hydrogen bond acceptors A1 and A2 and one aromatic ring R were the common part of two types of inhibitors. However, the hydrogen bond acceptor (A3) and donor (D) could well match in a large flat region (C site). The hydrophobic group (H) was an essential point in the B site. Thus, the refined pharmacophore was an ideal model that not only properly reflected the characterization of the PTP1B active site, but also contained binding modes between the inhibitors and PTP1B.

**Figure 10:**
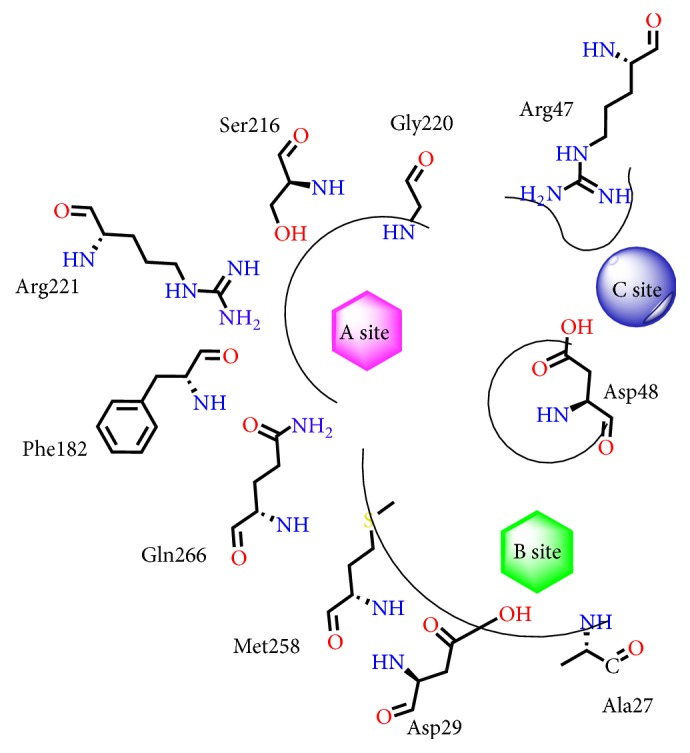
Schematic representation of the PTP1B active site. The A site was shown in purple, the B site was shown in green, and the C site was shown in blue.

Moreover, based on the structural information of PTP1B and docking results of ligands, three points could be proposed: (1) two hydrogen bond acceptors were key reasons why inhibitors bind to the A site with potent bioactivity; (2) an aromatic ring, adjacent to the two hydrogen bond acceptors, was also an essential pharmacophore; (3) the binding models of inhibitors depended on the linkers properties. The more flexible and small linkers could easier occupy the second phosphotyrosine binding site (B site). Otherwise, the compounds which have more rigid and large linkers could easier bind to a large flat region (C site). In conclusion, the perfect target-based common feature pharmacophore model involved three binding pockets A, B, and C and linkers (Figure 10).

## 4. Conclusion

In order to understand binding modes between PTP1B and 16 competitive inhibitors, we built a common feature pharmacophore model consisting of five chemical features (RAAHH): two hydrophobic groups H, an aromatic ring R, and two hydrogen bond acceptors A. Meanwhile, the characteristics of PTP1B active sites were depicted as three crucial regions, and the molecular docking was developed to reproduce experimental binding affinities for 16 inhibitors. To identify the docking accuracy about this target, native docking and crossing-docking simulations were evaluated by different docking programs. Interestingly, these docking results showed that a sole reference could not represent binding modes of all PTP1B inhibitors. Two PDB (codes: 2CMA and 2QBP) could be distinguished into two main groups based on their interactions between compounds and the PTP1B active site. Docking results were merged in the development of new pharmacophores, and two types of pharmacophores were obtained. Combination of the characteristics of the active site and docking results allowed us to weigh different binding patterns in the active sites. The two groups of pharmacophores were superimposed to obtain a refined pharmacophore (RHDAAA).

In a word, we identified that two hydrogen bond acceptors and an aromatic ring were essential anchoring points in the primary phosphate-binding pocket. In the C site, an additional hydrogen bond donor was located near Asp48 and played a pivotal role in binding affinity. Meanwhile, interactions with Met258 were essential for the cyclohexyl group of inhibitors in the B site. This led to the proposal of binding models inside three active sites of PTP1B: the A site involving three major chemical features: an aromatic ring and two hydrogen bond acceptors; the B site, a hydrophobic group as a secondary one; and a hydrogen bond donor well matched in the C site. In summary, target-based pharmacophore could be used soon as a database query to identify potential new PTP1B inhibitors and a meaningful model for PTP1B lead optimization.

## Supplementary Material

Figure S1: The binding pockets of the PTP1B, which is shown in surface mode labeled with training set: **1** (A), **2** (B), **3** (C), **4** (D), **5** (E), **6** (F), **7** (G), **8** (H), **9** (I), **10** (J), **11** (K), **12** (L), **13** (M), **14** (N), **15** (O) and **16** (P). Each compound was showed by different colors. Protein: hydrophobic residues (blue) and hydrophilic residues (red). The pictures were prepares using PyMol.
Figure S2: Docking of compounds **1** (A), **2** (B), **3** (C), **4** (D), **5** (E), **6** (F), **7** (G), **8** (H), **9** (I), **10** (J), **11** (K), **12** (L), **13** (M), **14** (N), **15** (O) and **16** (P) into the active site of PTP1B with key amino acid residues in all ligand binding poses. The key amino acid residues: nitrogen (blue), oxygen (red), carbon (green) and sulfur (gold). Compounds: nitrogen (blue), oxygen (red), carbon (white) and sulfur (gold). The pictures were prepares using PyMol.
Table S1: AutoDock 4, XP, and SP binding scores (kcal/mol) for docking studies of the training set.
Table S2: Non-bond interaction of each compound (training set) in PTP1B active sites.

## Figures and Tables

**Table 1:** Activities of the selected compounds.

Compound	IC_50_ (nM)	*K*_*i*_ (nM)	Ref.
**1**	210		[16]
**2**	185		[16]
**3**	65		[16]
**4**	110		[21]
**5**	330		[21]
**6**	31		[21]
**7**	7		[17]
**8**	90		[17]
**9**		36	[18]
**10**		4	[18]
**11**		13	[22]
**12**		740	[22]
**13**		310	[18]
**14**		21	[22]
**15**		22	[22]
**16**		0.68	[18]

**Table 2:** Summary of the pharmacophore models generated by HipHop for PTP1B inhibitors.

Hypothesis	Features	Rank	Direct hit	Partial hit	Max. fit
01	RNHHA	96.526	10111111	01000000	5
02	RHHAA	88.605	10111111	01000000	5
03	RNHHA	88.290	10111111	01000000	5
04	RRNAA	86.770	10111111	01000000	5
05	RNHA	85.881	11111111	00000000	4
06	RHHAA	85.819	10111111	01000000	5
07	RRHAA	85.193	10111111	01000000	5
08	RNHA	84.838	11111111	00000000	4
09	HHHAA	84.535	10111111	01000000	5
10	RHHAA	84.392	10111111	01000000	5
11	RNHA	83.968	11111111	00000000	4
12	RHHAA	83.877	10111111	01000000	5
13	RNHA	83.439	11111111	00000000	4
14	HHHAA	83.363	10111111	01000000	5
15	RHHAA	82.987	10111111	01000000	5

**Table 3:** Summary of the native docking in different software.

PDB	AutoDock Vina (kcal/mol)	AutoDock 4 (kcal/mol)	Glide (kcal/mol)		Xscore (kcal/mol)
			SP GlideScore	XP GlideScore	
2CM7	−5.80	−12.22	−8.28	−6.09	−9.18
2CMA	−7.00	−12.29	−8.69	−6.29	−9.30
2CMB	−8.00	−12.52	−10.28	−6.82	−9.82
2CNG	−9.30	−11.47	−8.02	−6.09	−9.55
2QBP	−7.50	−13.5	−10.18	−11.49	−9.82
2QBQ	−8.40	−12.13	−8.94	−9.45	−9.64
2ZN7	−8.10	−12.27	−9.77	−9.73	−9.24

**Table 4:** AutoDock 4, XP, and SP binding scores (kcal/mol) for docking studies of the training set.

Ligand	AutoDock 4		SP GlideScore		XP GlideScore	
	2CMA	2QBP	2CMA	2QBP	2CMA	2QBP
1	−12.05	−9.83	−8.90	−4.71	−8.87	−7.28
2	−12.37	−10.34	−8.69	−4.05	−8.15	−5.23
3	−12.43	−11.33	−10.00	−4.05	−10.40	−5.03
4	−10.97	−9.84	−8.66	−4.26	−8.88	−6.94
5	−11.96	−11.17	−9.06	−5.36	−9.15	−6.98
6	−13.07	−11.98	−8.47	−4.54	−9.61	−5.96
7	−10.37	−11.74	−7.33	−4.24	−6.72	−9.22
8	−8.02	−9.06	−8.11	−8.82	−6.23	−7.06
9	−9.15	−11.23	−5.82	−8.39	−8.31	−11.10
10	−9.91	−12.24	−5.55	−9.16	−7.37	−10.33
11	−9.64	−10.67	−5.32	−8.11	−3.89	−9.72
12	−9.11	−10.64	−5.96	−9.28	−7.24	−9.97
13	−8.74	−10.51	−5.38	−8.68	−6.90	−10.54
14	−8.98	−10.73	−5.56	−8.70	−7.07	−10.27
15	−8.99	−10.61	−5.14	−8.87	−7.19	−10.02
16	−9.85	−12.29	−5.86	−9.13	−8.12	−9.23
