# Supplementary material for: Computational Insight into Protein Tyrosine Phosphatase 1B Inhibition: A Case Study of the Combined Ligand- and Structure-Based Approach
Source: Comput Math Methods Med. 2017 Dec 26;2017:4245613. doi: 10.1155/2017/4245613 (PMC5758944; doi:10.1155/2017/4245613)
Supplement: Supplementary file 1 — Figure S1: The binding pockets of the PTP1B, which is shown in surface mode labeled with training set: 1 (A), 2 (B), 3 (C), 4 (D), 5 (E), 6 (F), 7 (G), 8 (H), 9 (I), 10 (J), 11 (K), 12 (L), 13 (M), 14 (N), 15 (O) and 16 (P). Each compound was showed by different colors. Protein: hydrophobic residues (blue) and hydrophilic residues (red). The pictures were prepares using PyMol. Figure S2: Docking of compounds 1 (A), 2 (B), 3 (C), 4 (D), 5 (E), 6 (F), 7 (G), 8 (H), 9 (I), 10 (J), 11 (K), 12 (L), 13 (M), 14 (N), 15 (O) and 16 (P) into the active site of PTP1B with key amino acid residues in all ligand binding poses. The key amino acid residues: nitrogen (blue), oxygen (red), carbon (green) and sulfur (gold). Compounds: nitrogen (blue), oxygen (red), carbon (white) and sulfur (gold). The pictures were prepares using PyMol. Table S1: AutoDock 4, XP, and SP binding scores (kcal/mol) for docking studies of the training set. Table S2: Non-bond interaction of each compound (training set) in PTP1B active sites. [file 4245613.f1.pdf]

Supplementary Material

Figure S1. The binding pockets of the PTP1B, which is shown in surface mode labeled with training set:**1** (A), **2** (B), **3** (C), **4** (D), **5** (E), **6** (F), **7** (G), **8** (H), **9** (I), **10** (J), **11** (K), **12** (L), **13** (M), **14** (N), **15** (O) and **16** (P). Each compound was showed by different colors. Protein: hydrophobic residues (blue) and hydrophilic residues (red). The pictures were prepares using PyMol.

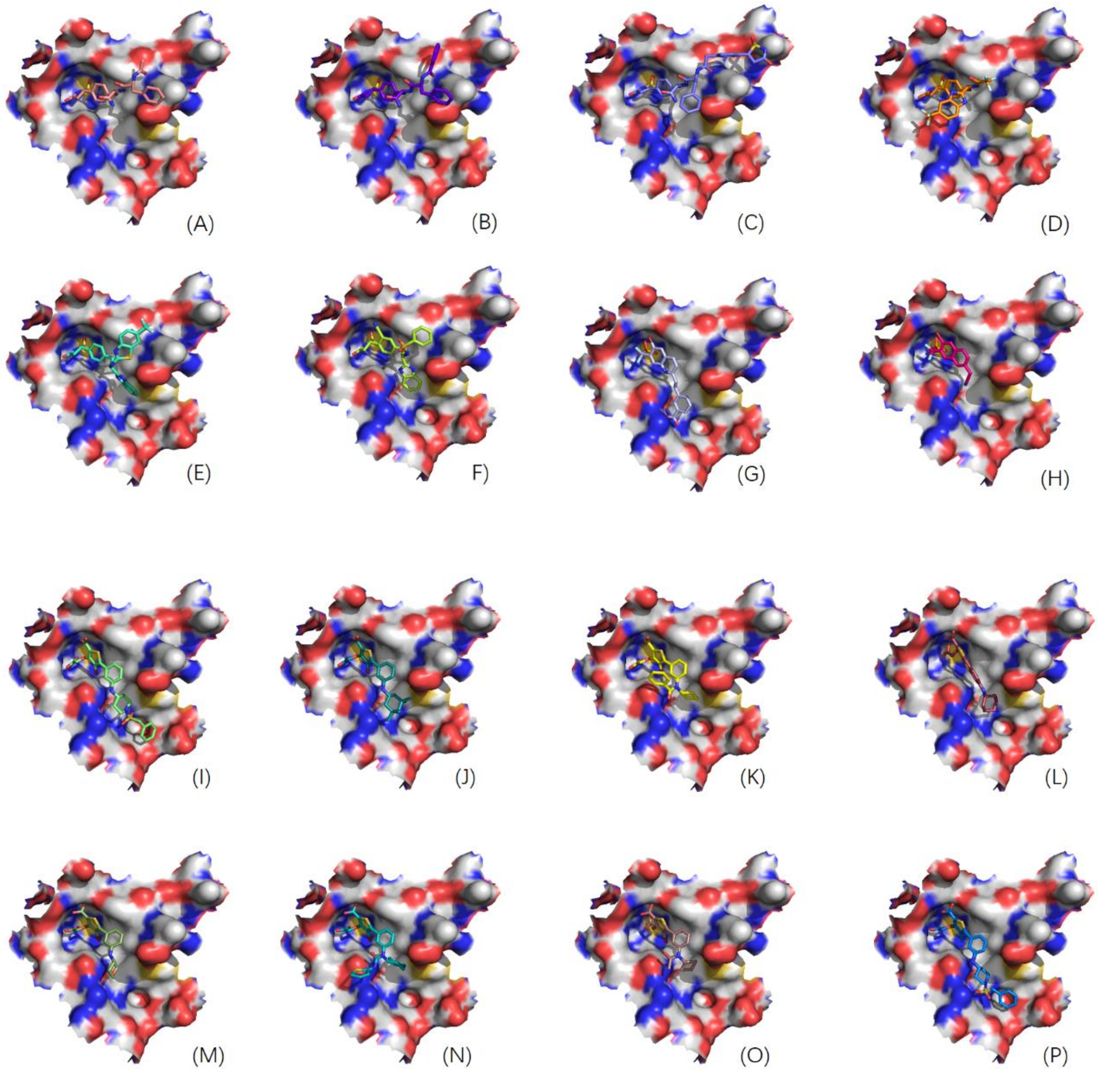

Figure S2. Docking of compounds **1** (A), **2** (B), **3** (C), **4** (D), **5** (E), **6** (F), **7** (G), **8** (H), **9** (I), **10** (J), **11** (K), **12** (L), **13** (M), **14** (N), **15** (O) and **16** (P) into the active site of PTP1B with key amino acid residues in all ligand binding poses. The key amino acid residues: nitrogen (blue), oxygen (red), carbon (green) and sulfur (gold). Compounds: nitrogen (blue), oxygen (red), carbon (white) and sulfur (gold). The pictures were prepares using PyMol.

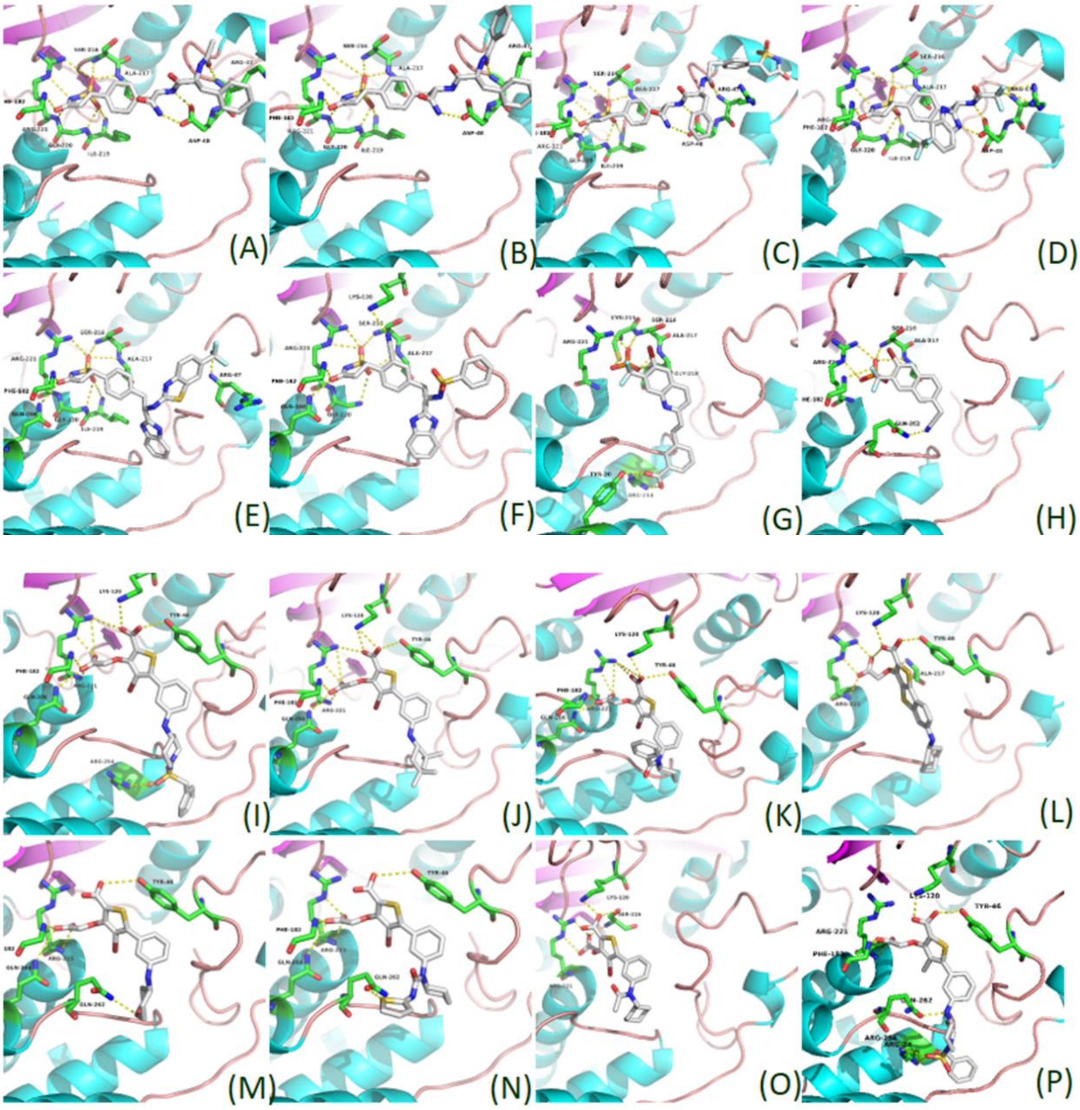

Table S1. AutoDock 4, XP, and SP binding scores (kcal/mol) for docking studies of the training set.

			autodock		SP Glide		XP Glide	
Compound	IC_{50} (nM)	Ki (nM)	2CMA	2QBP	2CMA	2QBP	2CMA	2QBP
1	210		-12.05	-9.83	-8.9	-4.71	-8.87	-7.28
2	185		-12.37	-10.34	-8.69	-4.05	-8.15	-5.23
3	65		-12.43	-11.33	-10	-4.05	-10.4	-5.03
4	110		-10.97	-9.84	-8.66	-4.26	-8.88	-6.94
5	330		-11.96	-11.17	-9.06	-5.36	-9.15	-6.98
6	31		-13.07	-11.98	-8.47	-4.54	-9.61	-5.96
7	7		-10.37	-11.74	-7.33	-4.24	-6.72	-9.22
8	90		-8.02	-9.06	-8.11	-8.82	-6.23	-7.06
9		36	-9.15	-11.23	-5.82	-8.39	-8.31	-11.1
10		4	-9.91	-12.24	-5.55	-9.16	-7.37	-10.33
11		13	-9.64	-10.67	-5.32	-8.11	-3.89	-9.72
12		740	-9.11	-10.64	-5.96	-9.28	-7.24	-9.97
13		310	-8.74	-10.51	-5.38	-8.68	-6.9	-10.54
14		21	-8.98	-10.73	-5.56	-8.7	-7.07	-10.27
15		22	-8.99	-10.61	-5.14	-8.87	-7.19	-10.02
16		0.68	-9.85	-12.29	-5.86	-9.13	-8.12	-9.23

Table S2. Non-bond interaction of each compound (training set) in PTP1B active sites.

Non-bond interaction of **Compound 1**

Name	Distance	Category	Types	From	From chemistry	To	To chemistry
d:RES1:S1 - A:ASP181:OD2	3.45464	Electrostatic	Attractive Charge	d:RES1:S1	Positive	A:ASP181:OD2	Negative
A:ARG47:HN - d:RES1:O34	1.76859	Hydrogen Bond	Conventional Hydrogen Bond	A:ARG47:HN	H-Donor	d:RES1:O34	H-Acceptor
A:SER216:HN - d:RES1:O14	1.91355	Hydrogen Bond	Conventional Hydrogen Bond	A:SER216:HN	H-Donor	d:RES1:O14	H-Acceptor
A:ALA217:HN - d:RES1:O13	2.30907	Hydrogen Bond	Conventional Hydrogen Bond	A:ALA217:HN	H-Donor	d:RES1:O13	H-Acceptor
A:GLY218:HN - d:RES1:O13	2.95127	Hydrogen Bond	Conventional Hydrogen Bond	A:GLY218:HN	H-Donor	d:RES1:O13	H-Acceptor
A:ILE219:HN - d:RES1:O13	2.69185	Hydrogen Bond	Conventional Hydrogen Bond	A:ILE219:HN	H-Donor	d:RES1:O13	H-Acceptor
A:GLY220:HN - d:RES1:O13	2.36031	Hydrogen Bond	Conventional Hydrogen Bond	A:GLY220:HN	H-Donor	d:RES1:O13	H-Acceptor
A:ARG221:HE - d:RES1:O14	2.83706	Hydrogen Bond	Conventional Hydrogen Bond	A:ARG221:HE	H-Donor	d:RES1:O14	H-Acceptor
A:ARG221:HH21 - d:RES1:O14	1.77613	Hydrogen Bond	Conventional Hydrogen Bond	A:ARG221:HH21	H-Donor	d:RES1:O14	H-Acceptor
A:GLN266:HE22 - d:RES1:O12	1.92299	Hydrogen Bond	Conventional Hydrogen Bond	A:GLN266:HE22	H-Donor	d:RES1:O12	H-Acceptor

d:RES1:H35 - A:CYS215:SG	2.80684	Hydrogen Bond	Conventional Hydrogen Bond	d:RES1:H35	H-Donor	A:CYS215:SG	H-Acceptor
d:RES1:H46 - A:ASP48:OD1	1.82346	Hydrogen Bond	Conventional Hydrogen Bond	d:RES1:H46	H-Donor	A:ASP48:OD1	H-Acceptor
d:RES1:H47 - A:ASP48:OD2	1.84741	Hydrogen Bond	Conventional Hydrogen Bond	d:RES1:H47	H-Donor	A:ASP48:OD2	H-Acceptor
A:ASP48:OD2 - d:RES1	3.69839	Electrostatic	Pi-Anion	A:ASP48:OD2	Negative	d:RES1	Pi-Orbitals
A:ALA217:CB - d:RES1	3.61934	Hydrophobic	Pi-Sigma	A:ALA217:CB	C-H	d:RES1	Pi-Orbitals
A:PHE182 - d:RES1	4.74972	Hydrophobic	Pi-Pi Stacked	A:PHE182	Pi-Orbitals	d:RES1	Pi-Orbitals
d:RES1 - A:ARG47	5.07219	Hydrophobic	Pi-Alkyl	d:RES1	Pi-Orbitals	A:ARG47	Alkyl

Non-bond interaction of **Compound 2**

Name	Distance	Category	Types	From	From chemistry	To	To chemistry
d:RES1:S1 - A:ASP181:OD2	3.60496	Electrostatic	Attractive Charge	d:RES1:S1	Positive	A:ASP181:OD2	Negative
A:ARG47:HN - d:RES1:O34	1.71344	Hydrogen Bond	Conventional Hydrogen Bond	A:ARG47:HN	H-Donor	d:RES1:O34	H-Acceptor
A:PHE182:HN - d:RES1:O12	2.66828	Hydrogen Bond	Conventional Hydrogen Bond	A:PHE182:HN	H-Donor	d:RES1:O12	H-Acceptor
A:SER216:HN - d:RES1:O14	1.76242	Hydrogen Bond	Conventional Hydrogen Bond	A:SER216:HN	H-Donor	d:RES1:O14	H-Acceptor
A:GLY218:HN - d:RES1:O13	2.95632	Hydrogen	Conventional	A:GLY218:H	H-Donor	d:RES1:O13	H-Acceptor

		Bond	Hydrogen Bond	N			
A:ILE219:HN - d:RES1:O13	2.57797	Hydrogen Bond	Conventional Hydrogen Bond	A:ILE219:HN	H-Donor	d:RES1:O13	H-Acceptor
A:GLY220:HN - d:RES1:O13	2.23505	Hydrogen Bond	Conventional Hydrogen Bond	A:GLY220:HN	H-Donor	d:RES1:O13	H-Acceptor
A:ARG221:HH21d:RES1:O14	2.05775	Hydrogen Bond	Conventional Hydrogen Bond	A:ARG221:HH21	H-Donor	d:RES1:O14	H-Acceptor
A:GLN266:HE22- d:RES1:O12	2.0268	Hydrogen Bond	Conventional Hydrogen Bond	A:GLN266:HE22	H-Donor	d:RES1:O12	H-Acceptor
d:RES1:H51 - A:ASP48:OD2	1.6607	Hydrogen Bond	Conventional Hydrogen Bond	d:RES1:H51	H-Donor	A:ASP48:OD2	H-Acceptor
d:RES1:H52 - A:ASP48:OD2	1.72351	Hydrogen Bond	Conventional Hydrogen Bond	d:RES1:H52	H-Donor	A:ASP48:OD2	H-Acceptor
A:ASP48:OD2 - d:RES1	3.3938	Electrostatic	Pi-Anion	A:ASP48:OD2	Negative	d:RES1	Pi-Orbitals
A:ALA217:CB - d:RES1	3.68542	Hydrophobic	Pi-Sigma	A:ALA217:CB	C-H	d:RES1	Pi-Orbitals
A:ARG45:O - d:RES1	2.9981	Other	Pi-Lone Pair	A:ARG45:O	Lone Pair	d:RES1	Pi-Orbitals
A:PHE182 - d:RES1	4.68612	Hydrophobic	Pi-Pi Stacked	A:PHE182	Pi-Orbitals	d:RES1	Pi-Orbitals

Non-bond interaction of **Compound 3**

Name	Distance	Category	Types	From	From chemistry	To	To chemistry
d:RES1:S1 -	3.718	Electro	Attractiv	d:RES	Positive	A:ASP181:	Negative

A:ASP181:OD2	23	static	e Charge	1:S1		OD2	
A:LYS36:HZ1 - d:RES1:O46	1.64071	Hydrogen Bond	Conventional Hydrogen Bond	A:LYS36:HZ1	H-Donor	d:RES1:O46	H-Acceptor
A:PHE182:HN - d:RES1:O12	2.37061	Hydrogen Bond	Conventional Hydrogen Bond	A:PHE182:HN	H-Donor	d:RES1:O12	H-Acceptor
A:SER216:HN - d:RES1:O14	2.37016	Hydrogen Bond	Conventional Hydrogen Bond	A:SER216:HN	H-Donor	d:RES1:O14	H-Acceptor
A:GLY218:HN - d:RES1:O13	2.58435	Hydrogen Bond	Conventional Hydrogen Bond	A:GLY218:HN	H-Donor	d:RES1:O13	H-Acceptor
A:ILE219:HN - d:RES1:O13	2.26439	Hydrogen Bond	Conventional Hydrogen Bond	A:ILE219:HN	H-Donor	d:RES1:O13	H-Acceptor
A:GLY220:HN - d:RES1:O13	2.11946	Hydrogen Bond	Conventional Hydrogen Bond	A:GLY220:HN	H-Donor	d:RES1:O13	H-Acceptor
A:ARG221:HH21 - d:RES1:O14	2.20283	Hydrogen Bond	Conventional Hydrogen Bond	A:ARG221:HH21	H-Donor	d:RES1:O14	H-Acceptor
A:GLN266:HE22 - d:RES1:O12	2.15748	Hydrogen Bond	Conventional Hydrogen Bond	A:GLN266:HE22	H-Donor	d:RES1:O12	H-Acceptor
d:RES1:O13 - A:CYS215:SG	2.61959	Hydrogen Bond	Conventional Hydrogen Bond	d:RES1:O13	H-Donor	A:CYS215:SG	H-Acceptor
d:RES1:O14 - A:ASP181:OD2	3.10197	Hydrogen Bond	Conventional Hydrogen Bond	d:RES1:O14	H-Donor	A:ASP181:OD2	H-Acceptor
d:RES1:H59 - A:ASP48:OD2	2.00914	Hydrogen Bond	Conventional Hydroge	d:RES1:H59	H-Donor	A:ASP48:OD2	H-Acceptor

			n Bond				
d:RES1:H62 - A:ASP48:OD2	2.75655	Hydrogen Bond	Conventional Hydrogen Bond	d:RES1:H62	H-Donor	A:ASP48:OD2	H-Acceptor
d:RES1:O46 - A:ARG47:O	2.73088	Hydrogen Bond	Conventional Hydrogen Bond	d:RES1:O46	H-Donor	A:ARG47:O	H-Acceptor
A:SER216:CB - d:RES1:O14	3.17211	Hydrogen Bond	Carbon Hydrogen Bond	A:SER216:CB	H-Donor	d:RES1:O14	H-Acceptor
A:ARG47:NH1 - d:RES1	4.37281	Electrostatic	Pi-Cation	A:ARG47:NH1	Positive	d:RES1	Pi-Orbitals
A:ASP48:OD2 - d:RES1	4.30795	Electrostatic	Pi-Anion	A:ASP48:OD2	Negative	d:RES1	Pi-Orbitals
A:ASP48:CB - d:RES1	3.41959	Hydrophobic	Pi-Sigma	A:ASP48:CB	C-H	d:RES1	Pi-Orbitals
A:PHE182 - d:RES1	5.24876	Hydrophobic	Pi-Pi Stacked	A:PHE182	Pi-Orbitals	d:RES1	Pi-Orbitals
d:RES1 - A:ALA217	3.34089	Hydrophobic	Pi-Alkyl	d:RES1	Pi-Orbitals	A:ALA217	Alkyl
d:RES1 - A:ILE219	5.41557	Hydrophobic	Pi-Alkyl	d:RES1	Pi-Orbitals	A:ILE219	Alkyl
d:RES1 - A:ARG47	4.73972	Hydrophobic	Pi-Alkyl	d:RES1	Pi-Orbitals	A:ARG47	Alkyl

Non-bond interaction of **Compound 4**

Name	Distance	Category	Types	From	From chemistry	To	To chemistry
d:RES1:S1 - A:ASP181:OD2	3.66699	Electrostatic	Attractive Charge	d:RES1:S1	Positive	A:ASP181:OD2	Negative
A:PHE182:HN - d:RES1:O12	2.19055	Hydrogen Bond	Conventional Hydrogen Bond	A:PHE182:HN	H-Donor	d:RES1:O12	H-Acceptor
A:SER216:HN - d:RES1:O14	1.71836	Hydrogen Bond	Conventional Hydrogen Bond	A:SER216:HN	H-Donor	d:RES1:O14	H-Acceptor

A:ALA217:HN - d:RES1:O14	2.09684	Hydrogen Bond	Conventional Hydrogen Bond	A:ALA217:HN	H-Donor	d:RES1:O14	H-Acceptor
A:GLY218:HN - d:RES1:O13	3.05106	Hydrogen Bond	Conventional Hydrogen Bond	A:GLY218:HN	H-Donor	d:RES1:O13	H-Acceptor
A:ILE219:HN - d:RES1:O13	2.44523	Hydrogen Bond	Conventional Hydrogen Bond	A:ILE219:HN	H-Donor	d:RES1:O13	H-Acceptor
A:GLY220:HN - d:RES1:O13	1.93814	Hydrogen Bond	Conventional Hydrogen Bond	A:GLY220:HN	H-Donor	d:RES1:O13	H-Acceptor
A:ARG221:HH21 - d:RES1:O14	2.27333	Hydrogen Bond	Conventional Hydrogen Bond	A:ARG221:HH21	H-Donor	d:RES1:O14	H-Acceptor
A:ARG254:HH21 - d:RES1:F36	2.38712	Hydrogen Bond; Halogen	Conventional Hydrogen Bond;Halogen (Fluorine)	A:ARG254:HH21	H-Donor;Halogen Acceptor	d:RES1:F36	H-Acceptor; Halogen
A:ARG254:HH22 - d:RES1:F34	2.4545	Hydrogen Bond; Halogen	Conventional Hydrogen Bond;Halogen (Fluorine)	A:ARG254:HH22	H-Donor;Halogen Acceptor	d:RES1:F34	H-Acceptor; Halogen
A:GLY259:HN - d:RES1:F36	1.88387	Hydrogen Bond; Halogen	Conventional Hydrogen Bond;Halogen (Fluorine)	A:GLY259:HN	H-Donor;Halogen Acceptor	d:RES1:F36	H-Acceptor; Halogen

A:GLN266:HE22 - d:RES1:O12	2.225	Hydrogen Bond	Conventional Hydrogen Bond	A:GLN266:HE22	H-Donor	d:RES1:O12	H-Acceptor
d:RES1:H48 - A:ASP48:OD2	1.71608	Hydrogen Bond	Conventional Hydrogen Bond	d:RES1:H48	H-Donor	A:ASP48:OD2	H-Acceptor
d:RES1:H49 - A:ASP48:O	2.49765	Hydrogen Bond	Conventional Hydrogen Bond	d:RES1:H49	H-Donor	A:ASP48:O	H-Acceptor
A:ASP48:OD2 - d:RES1:F29	3.38929	Halogen	Halogen (Fluorine)	A:ASP48:OD2	Halogen Acceptor	d:RES1:F29	Halogen
A:ASP48:OD2 - d:RES1:F30	3.34295	Halogen	Halogen (Fluorine)	A:ASP48:OD2	Halogen Acceptor	d:RES1:F30	Halogen
A:ARG254:CZ - d:RES1:F36	3.67026	Halogen	Halogen (Fluorine)	A:ARG254:CZ	Halogen Acceptor	d:RES1:F36	Halogen
A:ALA217:CB - d:RES1	3.56832	Hydrophobic	Pi-Sigma	A:ALA217:CB	C-H	d:RES1	Pi-Orbitals
A:MET258:SD - d:RES1	5.7646	Other	Pi-Sulfur	A:MET258:SD	Sulfur	d:RES1	Pi-Orbitals
A:MET258:SD - d:RES1	4.57383	Other	Pi-Sulfur	A:MET258:SD	Sulfur	d:RES1	Pi-Orbitals
A:PHE182 - d:RES1	4.82537	Hydrophobic	Pi-Pi Stacked	A:PHE182	Pi-Orbitals	d:RES1	Pi-Orbitals
d:RES1 - A:ILE219	5.24073	Hydrophobic	Pi-Alkyl	d:RES1	Pi-Orbitals	A:ILE219	Alkyl
d:RES1 - A:VAL49	5.26322	Hydrophobic	Pi-Alkyl	d:RES1	Pi-Orbitals	A:VAL49	Alkyl
d:RES1 - A:ILE219	5.47797	Hydrophobic	Pi-Alkyl	d:RES1	Pi-Orbitals	A:ILE219	Alkyl

Non-bond interaction of **Compound 5**

Name	Distance	Category	Types	From	From chemistry	To	To chemistry

d:RES1:S1 - A:ASP181:OD2	3.44447	Electrostatic	Attractive Charge	d:RES1:S1	Positive	A:ASP181:OD2	Negative
A:LYS120:HZ2 - d:RES1:F38	2.09617	Hydrogen Bond; Halogen	Conventional Hydrogen Bond;Halogen (Fluorine)	A:LYS120:HZ2	H-Donor;Halogen Acceptor	d:RES1:F38	H-Acceptor; Halogen
A:PHE182:HN - d:RES1:O12	2.46737	Hydrogen Bond	Conventional Hydrogen Bond	A:PHE182:HN	H-Donor	d:RES1:O12	H-Acceptor
A:SER216:HN - d:RES1:O14	1.83589	Hydrogen Bond	Conventional Hydrogen Bond	A:SER216:HN	H-Donor	d:RES1:O14	H-Acceptor
A:ALA217:HN - d:RES1:O13	2.41779	Hydrogen Bond	Conventional Hydrogen Bond	A:ALA217:HN	H-Donor	d:RES1:O13	H-Acceptor
A:GLY218:HN - d:RES1:O13	2.91872	Hydrogen Bond	Conventional Hydrogen Bond	A:GLY218:HN	H-Donor	d:RES1:O13	H-Acceptor
A:ILE219:HN - d:RES1:O13	2.59788	Hydrogen Bond	Conventional Hydrogen Bond	A:ILE219:HN	H-Donor	d:RES1:O13	H-Acceptor
A:GLY220:HN - d:RES1:O13	2.22276	Hydrogen Bond	Conventional Hydrogen Bond	A:GLY220:HN	H-Donor	d:RES1:O13	H-Acceptor
A:ARG221:HH21 - d:RES1:O14	1.77349	Hydrogen Bond	Conventional Hydrogen Bond	A:ARG221:HH21	H-Donor	d:RES1:O14	H-Acceptor
A:GLN266:HE22 - d:RES1:O12	1.92305	Hydrogen Bond	Conventional Hydrogen Bond	A:GLN266:HE22	H-Donor	d:RES1:O12	H-Acceptor
d:RES1:O13 - A:CYS215:SG	2.94181	Hydrogen	Conventional	d:RES1:O13	H-Donor	A:CYS215:SG	H-Acceptor

		Bond	Hydrogen Bond				
d:RES1:H51 - A:ASP48:OD2	2.07156	Hydrogen Bond	Conventional Hydrogen Bond	d:RES1:H51	H-Donor	A:ASP48:OD2	H-Acceptor
d:RES1:H52 - A:ASP48:OD2	2.06956	Hydrogen Bond	Conventional Hydrogen Bond	d:RES1:H52	H-Donor	A:ASP48:OD2	H-Acceptor
d:RES1:S28 - A:ASP48:OD1	3.23241	Other	Sulfur-X	d:RES1:S28	Sulfur	A:ASP48:OD1	O,N,S
d:RES1:S28 - A:ASP48:OD2	3.29681	Other	Sulfur-X	d:RES1:S28	Sulfur	A:ASP48:OD2	O,N,S
A:ASP48:OD1 - d:RES1	4.59065	Electrostatic	Pi-Anion	A:ASP48:OD1	Negative	d:RES1	Pi-Orbitals
A:VAL49:CG2 - d:RES1	3.82716	Hydrophobic	Pi-Sigma	A:VAL49:CG2	C-H	d:RES1	Pi-Orbitals
A:ALA217:CB - d:RES1	3.46346	Hydrophobic	Pi-Sigma	A:ALA217:CB	C-H	d:RES1	Pi-Orbitals
d:RES1:S28 - d:RES1	5.7457	Other	Pi-Sulfur	d:RES1:S28	Sulfur	d:RES1	Pi-Orbitals
A:PHE182 - d:RES1	4.83766	Hydrophobic	Pi-Pi Stacked	A:PHE182	Pi-Orbitals	d:RES1	Pi-Orbitals
d:RES1 - d:RES1	4.87603	Hydrophobic	Pi-Pi Stacked	d:RES1	Pi-Orbitals	d:RES1	Pi-Orbitals
A:TYR46 - d:RES1	4.68475	Hydrophobic	Pi-Pi T-shaped	A:TYR46	Pi-Orbitals	d:RES1	Pi-Orbitals
d:RES1 - A:TYR46	4.72465	Hydrophobic	Pi-Pi T-shaped	d:RES1	Pi-Orbitals	A:TYR46	Pi-Orbitals
d:RES1 - A:ILE219	5.33424	Hydrophobic	Pi-Alkyl	d:RES1	Pi-Orbitals	A:ILE219	Alkyl
d:RES1 - A:VAL49	5.4911	Hydrophobic	Pi-Alkyl	d:RES1	Pi-Orbitals	A:VAL49	Alkyl
d:RES1 - A:MET258	4.78396	Hydrophobic	Pi-Alkyl	d:RES1	Pi-Orbitals	A:MET258	Alkyl

Non-bond interaction of **Compound 6**

Name	Dista	Categ	Types	From	From	To	To

	nce	ory			chemistry		chemistry
d:RES1:S1 - A:ASP181:OD2	3.51772	Electrostatic	Attractive Charge	d:RES1:S1	Positive	A:ASP181:OD2	Negative
A:SER216:HN - d:RES1:O14	1.79274	Hydrogen Bond	Conventional Hydrogen Bond	A:SER216:HN	H-Donor	d:RES1:O14	H-Acceptor
A:GLY218:HN - d:RES1:O13	2.87658	Hydrogen Bond	Conventional Hydrogen Bond	A:GLY218:HN	H-Donor	d:RES1:O13	H-Acceptor
A:ILE219:HN - d:RES1:O13	2.61624	Hydrogen Bond	Conventional Hydrogen Bond	A:ILE219:HN	H-Donor	d:RES1:O13	H-Acceptor
A:GLY220:HN - d:RES1:O13	2.32658	Hydrogen Bond	Conventional Hydrogen Bond	A:GLY220:HN	H-Donor	d:RES1:O13	H-Acceptor
A:ARG221:HH21 - d:RES1:O14	1.92085	Hydrogen Bond	Conventional Hydrogen Bond	A:ARG221:HH21	H-Donor	d:RES1:O14	H-Acceptor
A:GLN266:HE22 - d:RES1:O12	2.04378	Hydrogen Bond	Conventional Hydrogen Bond	A:GLN266:HE22	H-Donor	d:RES1:O12	H-Acceptor
d:RES1:H48 - A:ASP48:OD2	1.7137	Hydrogen Bond	Conventional Hydrogen Bond	d:RES1:H48	H-Donor	A:ASP48:OD2	H-Acceptor
d:RES1:H49 - A:ASP48:OD2	2.18392	Hydrogen Bond	Conventional Hydrogen Bond	d:RES1:H49	H-Donor	A:ASP48:OD2	H-Acceptor
A:ASP48:OD1 - d:RES1	4.61338	Electrostatic	Pi-Anion	A:ASP48:OD1	Negative	d:RES1	Pi-Orbitals
A:VAL49:CG2 - d:RES1	3.81657	Hydrophobic	Pi-Sigma	A:VAL49:CG2	C-H	d:RES1	Pi-Orbitals
A:ALA217:CB - d:RES1	3.65662	Hydrophobic	Pi-Sigma	A:ALA217:C	C-H	d:RES1	Pi-Orbitals

				B			
d:RES1:S27 - A:PHE182	5.81846	Other	Pi-Sulfur	d:RES1:S27	Sulfur	A:PHE182	Pi-Orbitals
A:PHE182 - d:RES1	4.68702	Hydrophobic	Pi-Pi Stacked	A:PHE182	Pi-Orbitals	d:RES1	Pi-Orbitals
d:RES1 - d:RES1	4.52466	Hydrophobic	Pi-Pi Stacked	d:RES1	Pi-Orbitals	d:RES1	Pi-Orbitals
d:RES1 - A:VAL49	5.2504	Hydrophobic	Pi-Alkyl	d:RES1	Pi-Orbitals	A:VAL49	Alkyl
d:RES1 - A:MET258	4.69437	Hydrophobic	Pi-Alkyl	d:RES1	Pi-Orbitals	A:MET258	Alkyl

Non-bond interaction of **Compound 7**

Name	Distance	Category	Types	From	From chemistry	To	To chemistry
A:CYS215:HG - d:RES1:O17	1.98483	Hydrogen Bond	Conventional Hydrogen Bond	A:CYS215:HG	H-Donor	d:RES1:O17	H-Acceptor
A:SER216:HN - d:RES1:O18	2.40973	Hydrogen Bond	Conventional Hydrogen Bond	A:SER216:HN	H-Donor	d:RES1:O18	H-Acceptor
A:ALA217:HN - d:RES1:O16	2.05641	Hydrogen Bond	Conventional Hydrogen Bond	A:ALA217:HN	H-Donor	d:RES1:O16	H-Acceptor
A:GLY218:HN - d:RES1:O16	3.05522	Hydrogen Bond	Conventional Hydrogen Bond	A:GLY218:HN	H-Donor	d:RES1:O16	H-Acceptor
A:ARG221:HH22 - d:RES1:O18	1.76763	Hydrogen Bond	Conventional Hydrogen Bond	A:ARG221:HH22	H-Donor	d:RES1:O18	H-Acceptor
A:ARG254:HH11 - d:RES1:O28	1.7903	Hydrogen Bond	Conventional Hydrogen Bond	A:ARG254:HH11	H-Donor	d:RES1:O28	H-Acceptor
A:ARG254:HH	1.942	Hydro	Conventi	A:ARG	H-	d:RES1:O	H-

21 - d:RES1:O29	32	gen Bond	onal Hydrogen Bond	254:HH21	Donor	29	Acceptor
d:RES1:H42 - A:TYR20:OH	2.247	Hydrogen Bond	Conventional Hydrogen Bond	d:RES1:H42	H-Donor	A:TYR20:OH	H-Acceptor
A:SER216:CB - d:RES1:O18	2.8708	Hydrogen Bond	Carbon Hydrogen Bond	A:SER216:CB	H-Donor	d:RES1:O18	H-Acceptor
A:ALA217:CB - d:RES1	3.74162	Hydrophobic	Pi-Sigma	A:ALA217:CB	C-H	d:RES1	Pi-Orbitals
A:PHE182 - d:RES1	4.63959	Hydrophobic	Pi-Pi Stacked	A:PHE182	Pi-Orbitals	d:RES1	Pi-Orbitals
A:PHE182 - d:RES1	5.90562	Hydrophobic	Pi-Pi Stacked	A:PHE182	Pi-Orbitals	d:RES1	Pi-Orbitals
A:TYR46 - d:RES1	5.04555	Hydrophobic	Pi-Pi T-shaped	A:TYR46	Pi-Orbitals	d:RES1	Pi-Orbitals
A:TYR46 - d:RES1	5.11813	Hydrophobic	Pi-Pi T-shaped	A:TYR46	Pi-Orbitals	d:RES1	Pi-Orbitals
d:RES1:Br11 - A:LYS120	4.75497	Hydrophobic	Alkyl	d:RES1:Br11	Alkyl	A:LYS120	Alkyl
A:TYR46 - d:RES1:Br11	5.21328	Hydrophobic	Pi-Alkyl	A:TYR46	Pi-Orbitals	d:RES1:Br11	Alkyl
A:PHE182 - d:RES1:Br11	5.29224	Hydrophobic	Pi-Alkyl	A:PHE182	Pi-Orbitals	d:RES1:Br11	Alkyl
d:RES1 - A:VAL49	5.09428	Hydrophobic	Pi-Alkyl	d:RES1	Pi-Orbitals	A:VAL49	Alkyl
d:RES1 - A:ALA217	4.19749	Hydrophobic	Pi-Alkyl	d:RES1	Pi-Orbitals	A:ALA217	Alkyl

Non-bond interaction of **Compound 8**

Name	Distance	Category	Types	From	From chemistry	To	To chemistry
A: PHE182:HN - d:RES1:F15	2.88429	Hydrogen Bond; Halogen	Conventional Hydrogen Bond;Halogen	A:PHE182:HN	H-Donor;Halogen Acceptor	d:RES1:F15	H-Acceptor; Halogen

			(Fluorine)				
A:SER216:HN - d:RES1:O18	2.49643	Hydrogen Bond	Conventional Hydrogen Bond	A:SER216:HN	H-Donor	d:RES1:O18	H-Acceptor
A:ALA217:HN - d:RES1:O18	2.69875	Hydrogen Bond	Conventional Hydrogen Bond	A:ALA217:HN	H-Donor	d:RES1:O18	H-Acceptor
A:ALA217:HN - d:RES1:O16	2.57133	Hydrogen Bond	Conventional Hydrogen Bond	A:ALA217:HN	H-Donor	d:RES1:O16	H-Acceptor
A:ARG221:HE - d:RES1:O17	2.24031	Hydrogen Bond	Conventional Hydrogen Bond	A:ARG221:HE	H-Donor	d:RES1:O17	H-Acceptor
A:ARG221:HH22 - d:RES1:O18	2.15995	Hydrogen Bond	Conventional Hydrogen Bond	A:ARG221:HH22	H-Donor	d:RES1:O18	H-Acceptor
A:GLN262:HE22 - d:RES1:N21	2.59843	Hydrogen Bond	Conventional Hydrogen Bond	A:GLN262:HE22	H-Donor	d:RES1:N21	H-Acceptor
A:SER216:CB - d:RES1:O18	3.09853	Hydrogen Bond	Carbon Hydrogen Bond	A:SER216:CB	H-Donor	d:RES1:O18	H-Acceptor
A:ALA217:CB - d:RES1	3.77669	Hydrophobic	Pi-Sigma	A:ALA217:CB	C-H	d:RES1	Pi-Orbitals
A:PHE182 - d:RES1	4.63656	Hydrophobic	Pi-Pi Stacked	A:PHE182	Pi-Orbitals	d:RES1	Pi-Orbitals
A:TYR46 - d:RES1	4.79921	Hydrophobic	Pi-Pi T-shaped	A:TYR46	Pi-Orbitals	d:RES1	Pi-Orbitals
A:TYR46 - d:RES1	4.99751	Hydrophobic	Pi-Pi T-shaped	A:TYR46	Pi-Orbitals	d:RES1	Pi-Orbitals
d:RES1:Br11 - A:LYS120	4.9362	Hydrophobic	Alkyl	d:RES1:Br11	Alkyl	A:LYS120	Alkyl
A:PHE182 - d:RES1:Br11	4.99439	Hydrophobic	Pi-Alkyl	A:PHE182	Pi-Orbitals	d:RES1:Br11	Alkyl
d:RES1 - A:VAL49	5.03501	Hydrophobic	Pi-Alkyl	d:RES1	Pi-Orbitals	A:VAL49	Alkyl

d:RES1 - A:ALA217	4.1993	Hydrophobic	Pi-Alkyl	d:RES1	Pi-Orbitals	A:ALA217	Alkyl

Non-bond interaction of **Compound 9**

Name	Distance	Category	Types	From	From chemistry	To	To chemistry
A:PHE182:HN - d:RES1:O15	1.92464	Hydrogen Bond	Conventional Hydrogen Bond	A:PHE182:HN	H-Donor	d:RES1:O15	H-Acceptor
A:ARG221:HN - d:RES1:O14	1.98863	Hydrogen Bond	Conventional Hydrogen Bond	A:ARG221:HN	H-Donor	d:RES1:O14	H-Acceptor
A:ARG221:HE - d:RES1:O14	2.12791	Hydrogen Bond	Conventional Hydrogen Bond	A:ARG221:HE	H-Donor	d:RES1:O14	H-Acceptor
A:GLN266:HE21 - d:RES1:O15	2.78026	Hydrogen Bond	Conventional Hydrogen Bond	A:GLN266:HE21	H-Donor	d:RES1:O15	H-Acceptor
A:ALA217:CB - d:RES1	3.99195	Hydrophobic	Pi-Sigma	A:ALA217:CB	C-H	d:RES1	Pi-Orbitals
d:RES1 - A:TYR46	4.74613	Hydrophobic	Pi-Pi Stacked	d:RES1	Pi-Orbitals	A:TYR46	Pi-Orbitals
A:TYR46 - d:RES1	5.00148	Hydrophobic	Pi-Pi T-shaped	A:TYR46	Pi-Orbitals	d:RES1	Pi-Orbitals
A:VAL49 - d:RES1	5.29264	Hydrophobic	Alkyl	A:VAL49	Alkyl	d:RES1	Alkyl
A:ALA217 - d:RES1:Br7	3.40513	Hydrophobic	Alkyl	A:ALA217	Alkyl	d:RES1:Br7	Alkyl
d:RES1:Br7 - A:ILE219	4.13475	Hydrophobic	Alkyl	d:RES1:Br7	Alkyl	A:ILE219	Alkyl
d:RES1 - A:MET258	5.21694	Hydrophobic	Alkyl	d:RES1	Alkyl	A:MET258	Alkyl
d:RES1:C30 - A:ILE219	5.03211	Hydrophobic	Alkyl	d:RES1:C30	Alkyl	A:ILE219	Alkyl
d:RES1:C31 -	3.528	Hydro	Alkyl	d:RES	Alkyl	A:VAL49	Alkyl

A:VAL49	13	phobic		1:C31			
d:RES1:C31 - A:ILE219	4.071 41	Hydro phobic	Alkyl	d:RES 1:C31	Alkyl	A:ILE219	Alkyl
d:RES1:C31 - A:MET258	4.082 15	Hydro phobic	Alkyl	d:RES 1:C31	Alkyl	A:MET258	Alkyl
d:RES1 - A:VAL49	4.891 74	Hydro phobic	Pi-Alkyl	d:RES 1	Pi-Orbitals	A:VAL49	Alkyl
d:RES1 - A:ALA217	5.116 47	Hydro phobic	Pi-Alkyl	d:RES 1	Pi-Orbitals	A:ALA217	Alkyl

Non-bond interaction of **Compound 10**

Name	Dista nce	Categ ory	Types	From	From chemistr y	To	To chemistry
A:PHE182:HN - d:RES1:O15	1.900 44	Hydro gen Bond	Conventi onal Hydroge n Bond	A:PHE 182:H N	H-Donor	d:RES1:O 15	H-Acceptor
A:ARG221:HN - d:RES1:O14	1.983 59	Hydro gen Bond	Conventi onal Hydroge n Bond	A:ARG 221:H N	H-Donor	d:RES1:O 14	H-Acceptor
A:ARG221:HE - d:RES1:O14	1.991 6	Hydro gen Bond	Conventi onal Hydroge n Bond	A:ARG 221:H E	H-Donor	d:RES1:O 14	H-Acceptor
A:GLN266:HE2 1 - d:RES1:O15	2.750 06	Hydro gen Bond	Conventi onal Hydroge n Bond	A:GLN 266:H E21	H-Donor	d:RES1:O 15	H-Acceptor
A:ARG254:NH 1 - d:RES1	4.024 98	Electro static	Pi-Cation	A:ARG 254:N H1	Positive	d:RES1	Pi-Orbitals
A:ALA27:CB - d:RES1	3.641 67	Hydro phobic	Pi-Sigma	A:ALA 27:CB	C-H	d:RES1	Pi-Orbitals
d:RES1 - A:TYR46	4.830 57	Hydro phobic	Pi-Pi Stacked	d:RES 1	Pi-Orbitals	A:TYR46	Pi-Orbitals
A:TYR46 - d:RES1	4.832	Hydro phobic	Pi-Pi T-shaped	A:TYR 46	Pi-Orbitals	d:RES1	Pi-Orbitals
A:ALA217 - d:RES1:Br7	3.429 99	Hydro phobic	Alkyl	A:ALA 217	Alkyl	d:RES1:Br 7	Alkyl
d:RES1:Br7 - A:ILE219	4.182 32	Hydro phobic	Alkyl	d:RES 1:Br7	Alkyl	A:ILE219	Alkyl

d:RES1 - A:VAL49	5.03708	Hydrophobic	Pi-Alkyl	d:RES1	Pi-Orbitals	A:VAL49	Alkyl
d:RES1 - A:ALA217	5.10952	Hydrophobic	Pi-Alkyl	d:RES1	Pi-Orbitals	A:ALA217	Alkyl
d:RES1 - A:ALA217	4.0312	Hydrophobic	Pi-Alkyl	d:RES1	Pi-Orbitals	A:ALA217	Alkyl

Non-bond interaction of **Compound 11**

Name	Distance	Category	Types	From	From chemistry	To	To chemistry
A:PHE182:HN - d:RES1:O15	2.07063	Hydrogen Bond	Conventional Hydrogen Bond	A:PHE182:HN	H-Donor	d:RES1:O15	H-Acceptor
A:ARG221:HN - d:RES1:O14	1.98472	Hydrogen Bond	Conventional Hydrogen Bond	A:ARG221:HN	H-Donor	d:RES1:O14	H-Acceptor
A:ARG221:HE - d:RES1:O14	1.99269	Hydrogen Bond	Conventional Hydrogen Bond	A:ARG221:HE	H-Donor	d:RES1:O14	H-Acceptor
A:GLN266:HE21 - d:RES1:O15	2.40652	Hydrogen Bond	Conventional Hydrogen Bond	A:GLN266:HE21	H-Donor	d:RES1:O15	H-Acceptor
A:ARG24:NH2 - d:RES1	4.02255	Hydrogen Bond;Electrostatic	Pi-Cation;Pi-Donor Hydrogen Bond	A:ARG24:NH2	Positive;H-Donor	d:RES1	Pi-Orbitals;Pi-Orbitals
d:RES1 - A:TYR46	4.79478	Hydrophobic	Pi-Pi Stacked	d:RES1	Pi-Orbitals	A:TYR46	Pi-Orbitals
A:TYR46 - d:RES1	4.68565	Hydrophobic	Pi-Pi T-shaped	A:TYR46	Pi-Orbitals	d:RES1	Pi-Orbitals
A:VAL49 - d:RES1	5.46371	Hydrophobic	Alkyl	A:VAL49	Alkyl	d:RES1	Alkyl
A:ALA217 - d:RES1:Br7	3.44657	Hydrophobic	Alkyl	A:ALA217	Alkyl	d:RES1:Br7	Alkyl
d:RES1:Br7 - A:ILE219	4.23077	Hydrophobic	Alkyl	d:RES1:Br7	Alkyl	A:ILE219	Alkyl
d:RES1 -	5.069	Hydro	Pi-Alkyl	d:RES	Pi-	A:VAL49	Alkyl

A:VAL49	03	phobic		1	Orbitals		
d:RES1 - A:ALA217	5.08848	Hydrophobic	Pi-Alkyl	d:RES1	Pi-Orbitals	A:ALA217	Alkyl
d:RES1 - A:ALA217	4.00228	Hydrophobic	Pi-Alkyl	d:RES1	Pi-Orbitals	A:ALA217	Alkyl

Non-bond interaction of **Compound 12**

Name	Distance	Category	Types	From	From chemistry	To	To chemistry
A:LYS120:HZ1 - d:RES1:O8	1.97889	Hydrogen Bond	Conventional Hydrogen Bond	A:LYS120:HZ1	H-Donor	d:RES1:O8	H-Acceptor
A:ALA217:HN - d:RES1:O9	2.58127	Hydrogen Bond	Conventional Hydrogen Bond	A:ALA217:HN	H-Donor	d:RES1:O9	H-Acceptor
A:ARG221:HN - d:RES1:O13	1.97747	Hydrogen Bond	Conventional Hydrogen Bond	A:ARG221:HN	H-Donor	d:RES1:O13	H-Acceptor
A:ARG221:HE - d:RES1:O13	1.99251	Hydrogen Bond	Conventional Hydrogen Bond	A:ARG221:HE	H-Donor	d:RES1:O13	H-Acceptor
A:ARG221:HH22 - d:RES1:O12	1.82381	Hydrogen Bond	Conventional Hydrogen Bond	A:ARG221:HH22	H-Donor	d:RES1:O12	H-Acceptor
d:RES1:H31 - d:RES1:O8	2.744	Hydrogen Bond	Conventional Hydrogen Bond	d:RES1:H31	H-Donor	d:RES1:O8	H-Acceptor
d:RES1:H28 - A:TYR46:OH	2.11313	Hydrogen Bond	Conventional Hydrogen Bond	d:RES1:H28	H-Donor	A:TYR46:OH	H-Acceptor
A:SER216:CB - d:RES1:O9	3.64962	Hydrogen	Carbon Hydroge	A:SER216:C	H-Donor	d:RES1:O9	H-Acceptor

		Bond	n Bond	B			
A:SER216:CB - d:RES1:O8	3.32476	Hydrogen Bond	Carbon Hydrogen Bond	A:SER216:CB	H-Donor	d:RES1:O8	H-Acceptor
d:RES1:S5 - A:PHE182	4.47513	Other	Pi-Sulfur	d:RES1:S5	Sulfur	A:PHE182	Pi-Orbitals
d:RES1 - A:TYR46	4.59613	Hydrophobic	Pi-Pi Stacked	d:RES1	Pi-Orbitals	A:TYR46	Pi-Orbitals
d:RES1 - A:PHE182	4.59549	Hydrophobic	Pi-Pi T-shaped	d:RES1	Pi-Orbitals	A:PHE182	Pi-Orbitals
d:RES1 - A:MET258	4.92314	Hydrophobic	Alkyl	d:RES1	Alkyl	A:MET258	Alkyl
d:RES1 - A:ALA217	4.0488	Hydrophobic	Pi-Alkyl	d:RES1	Pi-Orbitals	A:ALA217	Alkyl
d:RES1 - A:VAL49	5.42147	Hydrophobic	Pi-Alkyl	d:RES1	Pi-Orbitals	A:VAL49	Alkyl
d:RES1 - A:ALA217	3.86353	Hydrophobic	Pi-Alkyl	d:RES1	Pi-Orbitals	A:ALA217	Alkyl
d:RES1 - A:ILE219	5.39874	Hydrophobic	Pi-Alkyl	d:RES1	Pi-Orbitals	A:ILE219	Alkyl
d:RES1 - A:VAL49	5.2762	Hydrophobic	Pi-Alkyl	d:RES1	Pi-Orbitals	A:VAL49	Alkyl

Non-bond interaction of **Compound 13**

Name	Distance	Category	Types	From	From chemistry	To	To chemistry
A:PHE182:HN - d:RES1:O15	1.91674	Hydrogen Bond	Conventional Hydrogen Bond	A:PHE182:HN	H-Donor	d:RES1:O15	H-Acceptor
A:ARG221:HN - d:RES1:O14	1.99523	Hydrogen Bond	Conventional Hydrogen Bond	A:ARG221:HN	H-Donor	d:RES1:O14	H-Acceptor
A:ARG221:HE - d:RES1:O14	2.0868	Hydrogen Bond	Conventional Hydrogen Bond	A:ARG221:HE	H-Donor	d:RES1:O14	H-Acceptor
A:GLN262:HE21 - d:RES1:O25	2.33166	Hydrogen Bond	Conventional Hydrogen	A:GLN262:HE21	H-Donor	d:RES1:O25	H-Acceptor

			n Bond				
A:GLN266:HE21 - d:RES1:O15	2.76024	Hydrogen Bond	Conventional Hydrogen Bond	A:GLN266:HE21	H-Donor	d:RES1:O15	H-Acceptor
d:RES1 - A:TYR46	4.78459	Hydrophobic	Pi-Pi Stacked	d:RES1	Pi-Orbitals	A:TYR46	Pi-Orbitals
A:TYR46 - d:RES1	4.96384	Hydrophobic	Pi-Pi T-shaped	A:TYR46	Pi-Orbitals	d:RES1	Pi-Orbitals
A:ALA217 - d:RES1:Br7	3.46536	Hydrophobic	Alkyl	A:ALA217	Alkyl	d:RES1:Br7	Alkyl
d:RES1:Br7 - A:ILE219	4.19782	Hydrophobic	Alkyl	d:RES1:Br7	Alkyl	A:ILE219	Alkyl
d:RES1 - A:ALA217	4.03234	Hydrophobic	Pi-Alkyl	d:RES1	Pi-Orbitals	A:ALA217	Alkyl
d:RES1 - A:VAL49	5.02382	Hydrophobic	Pi-Alkyl	d:RES1	Pi-Orbitals	A:VAL49	Alkyl
d:RES1 - A:ALA217	5.17236	Hydrophobic	Pi-Alkyl	d:RES1	Pi-Orbitals	A:ALA217	Alkyl

Non-bond interaction of **Compound 14**

Name	Distance	Category	Types	From	From chemistry	To	To chemistry
A:PHE182:HN - d:RES1:O15	1.88828	Hydrogen Bond	Conventional Hydrogen Bond	A:PHE182:HN	H-Donor	d:RES1:O15	H-Acceptor
A:ARG221:HN - d:RES1:O14	1.98085	Hydrogen Bond	Conventional Hydrogen Bond	A:ARG221:HN	H-Donor	d:RES1:O14	H-Acceptor
A:ARG221:HE - d:RES1:O14	1.98914	Hydrogen Bond	Conventional Hydrogen Bond	A:ARG221:HE	H-Donor	d:RES1:O14	H-Acceptor
A:GLN266:HE21 - d:RES1:O15	2.76689	Hydrogen Bond	Conventional Hydrogen Bond	A:GLN266:HE21	H-Donor	d:RES1:O15	H-Acceptor
d:RES1:S35 -	2.998	Other	Sulfur-X	d:RES	Sulfur	A:GLN262	O,N,S

A:GLN262:OE1	98			1:S35		:OE1	
A:ARG24:NH1 - d:RES1	3.81081	Hydrogen Bond;Electrostatic	Pi-Cation;Pi-Donor Hydrogen Bond	A:ARG24:NH1	Positive; H-Donor	d:RES1	Pi-Orbitals;Pi-Orbitals
A:ALA217:CB - d:RES1	3.9718	Hydrophobic	Pi-Sigma	A:ALA217:CB	C-H	d:RES1	Pi-Orbitals
d:RES1 - A:TYR46	4.81651	Hydrophobic	Pi-Pi Stacked	d:RES1	Pi-Orbitals	A:TYR46	Pi-Orbitals
A:TYR46 - d:RES1	4.9565	Hydrophobic	Pi-Pi T-shaped	A:TYR46	Pi-Orbitals	d:RES1	Pi-Orbitals
A:VAL49 - d:RES1	4.93612	Hydrophobic	Alkyl	A:VAL49	Alkyl	d:RES1	Alkyl
A:ALA217 - d:RES1:Br7	3.47762	Hydrophobic	Alkyl	A:ALA217	Alkyl	d:RES1:Br7	Alkyl
d:RES1:Br7 - A:ILE219	4.13567	Hydrophobic	Alkyl	d:RES1:Br7	Alkyl	A:ILE219	Alkyl
d:RES1 - A:VAL49	4.95365	Hydrophobic	Pi-Alkyl	d:RES1	Pi-Orbitals	A:VAL49	Alkyl
d:RES1 - A:ALA217	5.09512	Hydrophobic	Pi-Alkyl	d:RES1	Pi-Orbitals	A:ALA217	Alkyl

Non-bond interaction of **Compound 15**

Name	Distance	Category	Types	From	From chemistry	To	To chemistry
A:LYS120:HZ1 - d:RES1:O9	1.95185	Hydrogen Bond	Conventional Hydrogen Bond	A:LYS120:HZ1	H-Donor	d:RES1:O9	H-Acceptor
A:SER216:HN - d:RES1:O15	2.00908	Hydrogen Bond	Conventional Hydrogen Bond	A:SER216:HN	H-Donor	d:RES1:O15	H-Acceptor
A:ARG221:HE - d:RES1:O14	2.31403	Hydrogen Bond	Conventional Hydrogen Bond	A:ARG221:HE	H-Donor	d:RES1:O14	H-Acceptor
A:ARG221:HH22 -	1.82026	Hydrogen	Conventional	A:ARG221:H	H-Donor	d:RES1:O15	H-Acceptor

d:RES1:O15		Bond	Hydrogen Bond	H22			
A:SER216:CB - d:RES1:O15	3.06942	Hydrogen Bond	Carbon Hydrogen Bond	A:SER216:CB	H-Donor	d:RES1:O15	H-Acceptor
d:RES1:S5 - A:PHE182	4.84914	Other	Pi-Sulfur	d:RES1:S5	Sulfur	A:PHE182	Pi-Orbitals
A:TYR46 - d:RES1	5.19161	Hydrophobic	Pi-Pi T-shaped	A:TYR46	Pi-Orbitals	d:RES1	Pi-Orbitals
A:VAL49 - d:RES1	5.00326	Hydrophobic	Alkyl	A:VAL49	Alkyl	d:RES1	Alkyl
A:ALA217 - d:RES1:Br7	3.82901	Hydrophobic	Alkyl	A:ALA217	Alkyl	d:RES1:Br7	Alkyl
A:ILE219 - d:RES1	5.14882	Hydrophobic	Alkyl	A:ILE219	Alkyl	d:RES1	Alkyl
d:RES1:Br7 - A:ILE219	4.48091	Hydrophobic	Alkyl	d:RES1:Br7	Alkyl	A:ILE219	Alkyl
d:RES1 - A:VAL49	5.38975	Hydrophobic	Pi-Alkyl	d:RES1	Pi-Orbitals	A:VAL49	Alkyl
d:RES1 - A:ALA217	4.2058	Hydrophobic	Pi-Alkyl	d:RES1	Pi-Orbitals	A:ALA217	Alkyl

Non-bond interaction of **Compound 16**

Name	Distance	Category	Types	From	From chemistry	To	To chemistry
A:LYS120:HZ1 - d:RES1:O10	2.07912	Hydrogen Bond	Conventional Hydrogen Bond	A:LYS120:HZ1	H-Donor	d:RES1:O10	H-Acceptor
A:PHE182:HN - d:RES1:O15	1.97342	Hydrogen Bond	Conventional Hydrogen Bond	A:PHE182:HN	H-Donor	d:RES1:O15	H-Acceptor
A:ARG221:HN - d:RES1:O14	1.94096	Hydrogen Bond	Conventional Hydrogen Bond	A:ARG221:HN	H-Donor	d:RES1:O14	H-Acceptor
A:ARG254:HH11 - d:RES1:O37	2.07396	Hydrogen Bond	Conventional Hydrogen Bond	A:ARG254:HH11	H-Donor	d:RES1:O37	H-Acceptor

A:ARG254:HH21 - d:RES1:O37	3.05455	Hydrogen Bond	Conventional Hydrogen Bond	A:ARG254:HH21	H-Donor	d:RES1:O37	H-Acceptor
d:RES1:H38 - A:TYR46:OH	1.91662	Hydrogen Bond	Conventional Hydrogen Bond	d:RES1:H38	H-Donor	A:TYR46:OH	H-Acceptor
A:ALA217:CB - d:RES1	3.91784	Hydrophobic	Pi-Sigma	A:ALA217:CB	C-H	d:RES1	Pi-Orbitals
A:MET258:SD - d:RES1	5.4783	Other	Pi-Sulfur	A:MET258:SD	Sulfur	d:RES1	Pi-Orbitals
d:RES1 - A:TYR46	4.84159	Hydrophobic	Pi-Pi Stacked	d:RES1	Pi-Orbitals	A:TYR46	Pi-Orbitals
A:TYR46 - d:RES1	5.86068	Hydrophobic	Pi-Pi T-shaped	A:TYR46	Pi-Orbitals	d:RES1	Pi-Orbitals
A:ALA217 - d:RES1:Cl28	3.71556	Hydrophobic	Alkyl	A:ALA217	Alkyl	d:RES1:Cl28	Alkyl
d:RES1:Cl28 - A:ILE219	4.19694	Hydrophobic	Alkyl	d:RES1:Cl28	Alkyl	A:ILE219	Alkyl
d:RES1 - A:VAL49	4.91109	Hydrophobic	Pi-Alkyl	d:RES1	Pi-Orbitals	A:VAL49	Alkyl
d:RES1 - A:ALA217	5.31293	Hydrophobic	Pi-Alkyl	d:RES1	Pi-Orbitals	A:ALA217	Alkyl
d:RES1 - A:ALA27	4.3449	Hydrophobic	Pi-Alkyl	d:RES1	Pi-Orbitals	A:ALA27	Alkyl
